# Nanotechnology in emerging liquid biopsy applications

**DOI:** 10.1186/s40580-021-00263-w

**Published:** 2021-05-02

**Authors:** Despina P. Kalogianni

**Affiliations:** Department of Chemistry, University of Patras, Rio, 26504 Patras, Greece

**Keywords:** Nanoparticles, Nanomaterials, Gold nanoparticles, Graphene oxide, GO, Carbon dots, Quantum dots, MiRNA, Circulating tumor DNA, Circulating tumor cells, Exosomes

## Abstract

Liquid biopsy is considered as the most attractive alternative to traditional tissue biopsies. The major advantages of this approach lie in the non-invasive procedure, the rapidness of sample collection and the potential for early cancer diagnosis and real-time monitoring of the disease and the treatment response. Nanotechnology has dynamically emerged in a wide range of applications in the field of liquid biopsy. The benefits of using nanomaterials for biosensing include high sensitivity and detectability, simplicity in many cases, rapid analysis, the low cost of the analysis and the potential for portability and personalized medicine. The present paper reports on the nanomaterial-based methods and biosensors that have been developed for liquid biopsy applications. Most of the nanomaterials used exhibit great analytical performance; moreover, extremely low limits of detection have been achieved for all studied targets. This review will provide scientists with a comprehensive overview of all the nanomaterials and techniques that have been developed for liquid biopsy applications. A comparison of the developed methods in terms of detectability, dynamic range, time-length of the analysis and multiplicity, is also provided.

**Supplementary Information:**

The online version contains supplementary material available at 10.1186/s40580-021-00263-w.

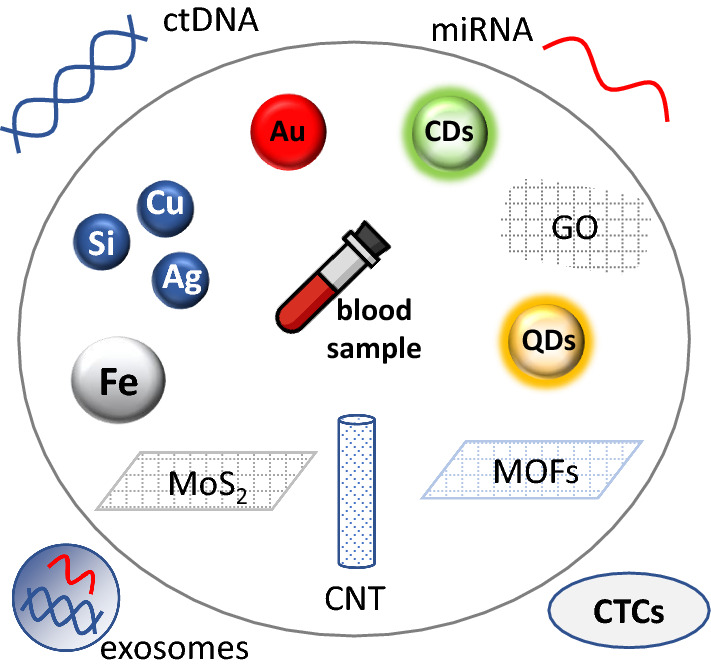

## Introduction

Nanotechnology-based methods have received wide attention, being the state-of-the-art among emerging technologies and promising alternatives to conventional methods. The major advantage of using nanotechnology in clinical diagnosis lies in the decrease of the cost of molecular profiling through the use of micro- and nanostructured sophisticated systems. Along with their rapid development and structural flexibility, micro/nanomaterials also constitute ideal miniaturized sensing platforms. Recent advances in nanomaterial synthesis have led to the development of novel sensitive, specific, and robust analytical tools [1].

The use of nanomaterials as labels/reporters in biosensing compared to conventional methods, yields significant advantages, such as high sensitivity, selectivity, stability, reproducibility, rapidness, portability, low-cost, large surface-to-volume ratio, ability for easy bioconjugation and point-of-care testing, along with the universal format and catalytic properties in many cases [2–6]. For fluorescence measurements, the use of fluorescent nanoparticles has eliminated the obstacles of photobleaching and photostability posed by common fluorescent dyes. Fluorescent nanoparticles have also proven to be an excellent and cost-effective alternative as fluorescence quenching platforms [7, 8]. Nanomaterials, such as carbon nanotubes and graphene oxide, have been utilized due to their high surface-to-volume ratio in order to overcome the insufficient capture efficiency and low purity of conventional methods for CTCs and exosomes detection. Graphene oxide and other nanostructures provide excellent solid supports for high-density immobilization of probes, enhancing detectability. A further major advantage of nanomaterials is their flexibility in synthesis, modification and bioconjugation [9]. Furthermore, nanoparticles have increased binding kinetics relative to other solid supports. This has led to rapid target enrichment and rapid analysis [10]. Finally, they have also been exploited for electrode modifications because of their large surface area and high electroconductivity, enhancing the immobilization of the biorecognition elements and accelerating the electron transfer for signal increasement [11].

Nanomaterials have been exploited in a plethora of applications. Nanomaterial-based applications are rapidly expanding from nanomedicine to food preservation, food packaging and fabric industry. Nanomaterials have been exploited in medicine, pharmacology, cosmetics, engineering, photonics, electronics and in food industry as antimicrobial agents, as well as in several sensing systems and in tumor and tissue imaging [12–14]. Another interesting application is that novel nanostructures can serve as excellent nanocarriers for drug delivery. Compared to common drugs, nanoparticles can be passively delivered to the tumor, prolonging the retention time of the drugs that they carry. Their easy conjugation to various ligands has also enhanced therapeutic efficacy [15]. Moreover, nanomaterials are good candidates for drug delivery even to the most complicated organs, such as the brain. Their specific advantages in this respect relate to the delivery of hydrophobic drugs, enhanced therapeutic efficacy, controlled drug dose and release at a specific location, which eliminates the toxic effects of the drugs [16]. As a result, there are numerous successful applications of nanoparticles both in cancer diagnostics and therapeutics and in the combination of these two (theranostics) [17] and several deliverable nano-systems, such as graphene oxide, gold nanoparticles, MnO_2_ nanoparticles and metal–organic-frameworks, have been successfully utilized for intracellular detection [18]. Moreover, gold nanoparticles, graphene oxide, carbon nanotubes and carbon dots, quantum dots, silver, silica and magnetic nanoparticles have been used for the detection of various biomarkers in body fluids (liquid biopsy) delivering significant improvements in clinical diagnostics. Finally, there has recently been a shift,in the synthesis of nanomaterials towards eco-friendly green synthesis methods. Several types of plants or microbes were used in such applications as natural reducing agents of metal precursors substances. These methods demonstrate large-scale potential and are considered as more inexpensive than chemical ones [13, 14, 19].

Liquid biopsies, on the other hand, have received special attention for cancer treatment and treatment monitoring. Early diagnosis of cancer is a major public health concern. Liquid biopsy has a very wide potential in tumor identification, optimization and monitoring. It offers a non-invasive, easy, quick and convenient alternative approach for early diagnosis, real-time monitoring of tumor progression, treatment progress, response and residual disease detection. Liquid biopsies have the potential to target various biomarkers (nucleic acids and proteins) for early cancer detection and provide guidance treatment. Liquid biopsy is mainly represented by blood circulating tumor cells (CTCs), circulating tumor DNA (ctDNA), circulating microRNAs (miRNAs) and exosomes that are secreted by the tumors. Using body fluid samples, liquid biopsies may constitute the ideal approach for the detection of these biomarkers or the products of primary or metastatic tumors, hence being a key factor for precision oncology. The aforementioned molecules may be secreted by multiple tumor sites, therefore liquid biopsy is expected to provide more valid information than invasive tissue biopsy. Additionally, monitoring of the disease can enable clinicians to adopt the optimal therapeutic strategy. Early detection and diagnosis have been a major issue in cancer research for many years, and the development of an easy and non-invasive detection method from a blood sample has been the aim of persistent effort [20–24]. The quantitative analysis of cancer biomarkers, such as miRNA or ctDNA, CTCs and exosomes, poses an analytical challenge because of their low molecular weight and extremely low abundance in blood circulation. For these reasons, enhancers are required for the analysis. Moreover, nucleic analysis also faces the challenge of ctDNA fragmentation and miRNA instability. Quantitative analysis is crucial for early diagnosis. Conventional methods, including DNA microarrays, reverse transcription quantitative PCR, next-generation sequencing, enzyme-linked immunosorbent assay (ELISA) and northern/southern and western blotting, cannot always meet this challenge due to their low sensitivity, selectivity and detectability, expensive instrumentation, high-cost analysis, as well as extensive sample pretreatment and the need for highly-trained personnel. As a result of these drawbacks, their application and/or rapid analysis is limited. DNA sequencing is complicated, too expensive, requires a rather lengthy period of time for the results (2–3 weeks) and may provide unnecessary data, while PCR-based amplification techniques require enzymatic amplification that may lead to amplification artifacts and the use of labels. DNA microarrays offer extremely high-throughput, but lack in sensitivity. In selectivity and specificity [21–29]. Furthermore, most of the techniques used are time-consuming, need large sample volume and lack practicality, such as high-throughput, simplicity, and multiplexing capacity. In addition, the most commonly used technique for exosomes’ detection is ultra-centrifugation up to 120,000 *g*, which is also time-consuming (> 10 h) and inefficient [30]. For these reasons, the aforementioned biomarkers have not yet been screened in a large population, delaying their use in common clinical practice. The analytical methods for screening and quantification of these molecules have still to be improved and rapid, simpler, and cost-effective approaches have to be developed [31, 32]. Signal amplification techniques have been introduced as substitutes for the above-mentioned conventional methods [28]. Nanomaterials have been significantly valuable in liquid biopsy applications for signal enhancement, as they do not require use of enzymatic reactions or multiple enhancement steps. Signal enhancement is mainly attributed to their high surface-to-volume ratio and their excellent optical and electrical properties [26]. The typical approach to improve the detectability of a sensor is to increase the surface-to-volume ratio along with the improved signal activity [33]. Contrariwise, enzymatic reactions are strongly affected by the environmental media, so their applications are limited because of long reaction times, specific reaction conditions, low reproducibility, and high cost, while their activity may be insufficient in complex biological samples [29–31, 34].

Previous literature reports include micro/nanomaterial-based systems for multiomics technologies and precision oncology [1], an overview of the existing liquid biopsy technologies in general [2], nanoarchitecture frameworks for electrochemical miRNA detection [4], nanotechnology-based liquid biopsy applications for ctDNA and exosomes detection [10], nucleic acids sensors for liquid biopsy applications [25] and plasmonic and supermagnetic nanomaterials for liquid biopsy applications [24, 35]. The present review reports on all the nanomaterials used in emerging liquid biopsy applications, targeting all the relevant biomarkers; furthermore, the analytical performance of the nanomaterials is also evaluated. The reported methods include electrochemical, electrochemiluminescent, fluorescent, colorimetric, optical and various spectrometric techniques. Finally, most of the reports have been applied for real-sample analysis or analysis in a real-sample environment.

## Signal enhancement approaches in liquid biopsy applications

The biomarkers targeted in liquid biopsy applications were various microRNAs molecules, circulating tumor double stranded-DNA (ctDNA) that contains specific tumor-related single nucleotide polymorphisms (SNPs), circulating tumor cells (CTCs) and exosomes, i.e., nano-vehicles secreted from the tumors that enter the blood circulation. The detection and quantification of these biomarkers form a challenge due to their low amount in body fluids, the possibility of ctDNA fragmentation and instability of miRNA and RNA molecules. DNA sequencing is one of the most frequently used techniques in liquid biopsy applications; however, there is still a need for developing lower-cost and faster methods or devices that will provide increased portability, practicality, sensitivity and specificity, as well as the potential of multiplex analysis and point-of-care testing [21, 22]. Nanomaterials have been exploited in liquid biopsy applications for signal improvement, due to the extremely low amounts of cancer ‘signature’ molecules present in body fluid samples. In many cases, however, other sophisticated signal enhancement ‘tricks’ were applied to further increase detectability. These ‘tricks’ are based either on target recycling—aiming to produce a lot of molecules-reporters—or signal amplification via different approaches. Target recycling is usually based on the specific function of DNAzymes-deoxyribozymes that exhibit catalytic action, duplex-specific nucleases (DSN) that degrade DNA strands in DNA-RNA hybrids and have no preference to single-stranded DNA (ssDNA) or RNA,nicking endonucleases that cleave specifically double-stranded DNA (dsDNA) in the presence of Mg^2+^ and strand displacement amplification (SDA) by other nucleic acid sequences/probes. Signal amplification depends mainly on hybridization chain reaction (HCR) or catalytic-hairpin assembly (CHA), rolling circle amplification (RCA), common target amplification strategies, or the use of metal nanoparticles to increase the surface-enhanced Raman spectroscopy (SERS), surface plasmon resonance (SPR) or the electrochemical signal [36, 37].

## Nanomaterials used in liquid biopsy applications

### Gold nanoparticles (AuNPs)

Gold nanoparticles are the most extensively used nanoparticles in liquid biopsy applications and numerous other applications. Their unique properties, such as optical and electrical properties, large surface to volume ratio, the capability of simple conjugation to several biomolecules, as well as their stability and biocompatibility have made them ideal for biosensor development [3]. Gold nanoparticles exhibit various properties, such as optical properties that allow for detection by naked-eye, electrical, surface plasmon resonance, and fluorescence resonance energy transfer (FRET), which have made them ideal for sensing in a plethora of different applications [32]. Several analytical methods have been reported for the detection of various biomarkers, including electrochemical, surface plasmon resonance spectroscopy (SPR), surface-enhanced Raman spectroscopy (SERS), fluorescence spectroscopy and colorimetric methods, lateral flow assays and other biosensors.

#### MicroRNAs

Gold nanoparticles have been widely used for the detection of various microRNA (miRNA) molecules that have been related to cancer emergence. In a recent report, DNA probes were conjugated to AuNPs and upon hybridization of miRNA target and in the presence of Mg^2+^, the produced DNAzyme cleaves the hybrid producing fluorescent fragments. The method gave an LOD of 50 fM [32]. An interesting approach involves the electron charging and discharging of Au cations via electron transfer from CdTe quantum dots (QDs) to AuNPs under UV light irradiation. Due to surface plasmon resonance, a change in their absorbance is caused, as the red solution of AuNPs turns into colorless. When miRNA target is hybridized to the DNA probes coupled to AuNPs, aggregation and fluorescence quenching of QDs takes place, hindering the above phenomenon. The method gave an LOD of 4.4 pM [38]. Moreover, a dual amplification system involved i) amplification of a DNA fragment after hybridization to the target miRNA and ii) the use of a nicking enzyme producing multiple DNA fragments that trigger a catalytic hairpin assembly (CHA) for signal amplification. Both reactions induce the aggregation of AuNPs which is monitored by absorbance measurements. The method had an LOD of 3.1 fM [39]. A colorimetric method was also developed by Nossier et al. (2018) for miRNA detection. The method was based on the stabilization of gold nanoparticles in the presence of high-salt concentration induced by the hybridization of miRNA target to a short complementary DNA probe. The method had an LOD of 330 nM (10 pmol) [40]. Positively charged gold nanoparticles were also used for colorimetric detection of miRNAs. Target miRNA, hybridized to complementary hairpins attached to gold nanoparticles, induced aggregation and a color change of the nanoparticle solution. The method offered an LOD of 100 aM [41].

Lateral flow tests (strips) were also used for miRNA detection using AuNPs as reporters for visual detection. The main advantages of these biosensors are the simplicity and the rapid analysis within few minutes. The assay is usually based on a sandwich-type hybridization assay on the nitrocellulose membrane providing an LOD of 7 pM [29]. Moreover, rolling circle amplification (RCA) in combination with AuNPs for dual signal enhancement and a lateral flow strip was used for simultaneous detection of 2 miRNAs with an LOD of 20 and 40 pM, respectively [42]. In another application, AuNPs were decorated with a detection probe and several horseradish peroxide (HPR) molecules that enhanced the optical readout upon addition of a chromogenic substrate. The method offered an LOD of 7.5 pM [43].

**Fig. 1:**
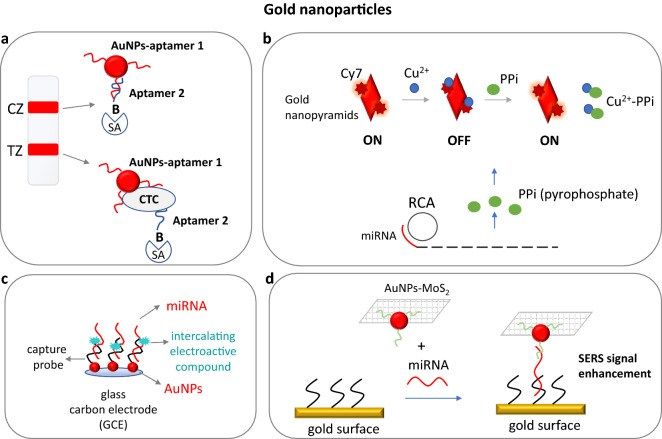
**a** An aptamer-AuNPs strip biosensor for visual detection of circulating tumor cells (CTCs). **b** Silica-coated gold nanobipyramids in combination with the fluorescent dye Cy7, which fluorescence is quenched by Cu^2+^ ions and recovered by pyrophosphates (PPi) that are produced during rolling circle amplification (RCA) reaction triggered by miRNA target. **c** an electrochemical detection of miRNA captured onto an electrode by deposited AuNPs-DNA. The signal is generated by an intercalating redox dye. **d** AuNPs decorated with molybdenum sulfide (MoS_2_) nanosheets are used for surface-enhanced Raman spectroscopy (SERS) signal enhancement in miRNA analysis. *AuNPs* gold nanoparticles, *TZ* test zone, *CZ* control zone, *SA* streptavidin, *B* biotin

Electrochemical biosensors based on AuNPs, have experienced good detectability, low cost, easy functionality, and good selectivity. A three-way junction RNA structure was designed containing a methylene blue-modified hairpin structure at its one leg to function as the sensing moiety, while the other two legs were hybridized to DNA-barcoded AuNPs for signal amplification. Hybridization of target miRNA resulted in opening of the hairpin moiety with subsequent hybridization onto a DNA-modified gold nanoflower/platinum electrode, leading to methylene blue oxidation. The method required a very low sample volume of 4 μL and offered an LOD of 135 aM that was 230 times higher than quantitative RT-PCR. Also, it gave wider dynamic range than quantitative RT-PCR, probably due to interferences of the serum samples on the enzymes used in the RT-PCR reaction [28]. Another approach reported the deposition of MoS_2_/g-C_3_N_4_/TiO_2_ on an indium tin oxide (ITO) electrode to increase the surface area, where DNA probe-AuNPs were assembled for extra signal enhancement. Target miRNA was then hybridized to the DNA probe. The hybrid was captured by a specific antibody, which was further interacted with secondary IgG antibodies coupled to AuNPs, leading to immobilization of horseradish peroxidase (HRP). HRP finally catalyzed the oxidation of its substrate, producing an insoluble product on the electrode’s surface and causing a decrease in the photocurrent. The developed biosensor had a detection limit of 0.13 fM [44]. A photoactive material, graphdiyne decorated with AuNPs was synthesized for miRNA detection. An RNA probe attached to this platform was hybridized to miRNA, while a second biotinylated RNA probe resulted in a sandwich-type structure. A streptavidin–alkaline phosphatase conjugate was then added to catalyze the formation of ascorbic acid from ascorbic acid 2-phosphate producing a photoelectrochemical response. The LOD of this sensor was **0.33 aM** [45]. Zhu et al. (2018) developed an electrochemical sensor for miRNA detection. MiRNA target was dually hybridized to complementary DNA probes immobilized onto the electrode’s surface and to gold nanostructures. Another DNA probe was attached to the gold nanostructures triggering an HCR, upon addition of target miRNA. The detection was finally accomplished through the electroactive compound Ru(NH3)_6_^3+^. The detection limit of the method was 0.12 fM of synthetic miRNA target [26]. Alternatively, in another approach, target miRNA triggered a target chain displacement polymerization reaction by DNA Klenow fragment, while the electrochemiluminescence signal was generated by a DNA/Ru(bpy)_3_^2+^/AuNPs complex, leading to a great signal enhancement with an LOD of 43 aM [46]. Target miRNA was also detected when it was captured by immobilized DNA probes onto gold nanorods on TiO_2_/ITO electrodes. The electrical signal was generated by biotinylated alkaline phosphatase and L-ascorbic acid 2-phosphate substrate oxidation producing the electroactive ascorbic acid. The method had an LOD of 2 nM [47]. Another HCR-based method was developed by Xiang et al. (2014). Target miRNA was hybridized, on an electrode’s surface, to gold nanoparticles coupled to a hairpin-like capture probe that contained an RNA sequence complementary to the target sequence. An HCR was initiated after cleavage of the RNA-RNA hybrid by ribonuclease A, releasing the miRNA target for target recycling. After HCR, G-quadruplex regions were formed, while the addition of hemin led to hemin/G-quadruplex complexes and gave an amplified electrochemical signal measured by differential pulse voltammetry. The LOD of the method was 100 fM [48]. The hybridization of a microRNA target to its complementary probe coupled to AuNPs onto an electrode’s surface was also monitored electrochemically using an intercalating redox dye, and offering a very low LOD of 78 aM (Fig. 1) [49]. Another electrochemiluminescence method was developed by Liu et al. (2017) based on the hybridization of the target miRNA to a molecular beacon labeled with Ru(bpy)_3_^2+^ and attached to AuNPs on an electrode’s surface with a low LOD of 10 fM [50]. Moreover, a triple signal amplification reaction through target-triggered cyclic duplex specific nuclease (DSN) digestion, bridge DNA − AuNPs and Ru(NH_3_)_6_^3+^ electroactive label was achieved for the detection of 6.8 aM of a miRNA sequence [51]. A voltametric detection of miRNA target was presented by Fredj et al. (2017). The target was hybridized to a biotinylated beacon attached to gold nanoparticles that was then captured onto a neutravidin coated electrode via avidin–biotin interaction. After signal enhancement, the authors were able to detect as low as 4 fM of miRNA target [52]. Another application involved signal amplification by DSN that cleaved the double-stranded hybrid between the miRNA target and a complementary hairpin probe. Short DNA fragments where produced and miRNA was released to initiate another cycle of DSN digestion. The DNA fragments were the captured by AuNPs conjugated to HRP or bare AuNPs on an electrodes surface enabling the detection of as low as 43.3 aM or 0.17 pM of miRNA, respectively [53, 54].

Gold nanoparticles in combination with strand displacement amplification (SDA) and magnetic beads were exploited in miRNA analysis with an LOD of 13.5 fM. More specifically, AuNPs were conjugated to a DNA probe complementary to the miRNA sequence and coupled to magnetic beads through a sandwich-type hybridization. MiRNA target was hybridized to its complementary probe through strand displacement reaction and led to the release of AuNPs from the magnetic beads. After the easy isolation of the laterals, the remaining AuNPs were measured using dark-field microscope and their concentration was related to the amount of the target [55].

A multiplex miRNA surface plasmon resonance (SPR) sensing system involved the hybridization of target miRNA to complementary DNA probes immobilized on the sensor’s surface. The hybrids were then detected by a biotinylated antibody that recognized DNA-RNA complexes, while neutravidin-AuNPs were used for signal enhancement. The sensor had an LOD of 0.5 pM [31]. Single-molecule detection of miRNA was accomplished through light-driven nano-oscillators. In more detail, a complementary to miRNA target DNA probe was dually attached onto the gold surface of an SPR sensor and to gold nanoparticles to enhance the SPR signal. The hybridization of miRNA target prevented the free oscillation of the DNA probe close to the gold’s surface of the sensor causing a change in the signal [56]. SPR techniques were also exploited in a simple combination with AuNPs and hybridization of unamplified miRNA to the surface of the SPR sensor or an optical fiber, offering an LOD of 500 pM and 0.27 pM, respectively [57, 58]. An effort was also made by Wu et al. (2019) and Zeng et al. (2017) to enhance the SPR signal. More specifically, an SPR sensor was based on dual signal amplification including SDA and AuNPs, succeeding an LOD of 0.5 fM [59]. Alternatively, the miRNA target was hybridized to a molecular beacon and amplified by a DNA polymerase. Then, a nicking enzyme recognized and cleaved the double-stranded hybrid, producing small DNA sequences (triggers) that triggered another amplification cycle. The growing number of DNA triggers greatly amplified the SPR signal for the detection of 45 pM of miRNA [60]. The use, however, of gold nanocubes enabled the detection of 5 pM of target miRNA [61]. Moreover, AuNPs decorated with molybdenum sulfide nanosheets lowered the detection limit down to 0.5 fM (Fig. 1) [62], while the combination of silver coated gold nanorods with HCR and colorimetric detection offered an LOD of 50 aM and the use of gold nanoprisms gave an LOD of 32.6 aM in buffer and 91 aM in human plasma [63, 64].

Surface-enhanced Raman spectroscopy (SERS) based on the combination of gold nanopilows or gold nanobowls with silver nanostructures and the dye Cy3 as the Raman substrate or AuNPs with methylene blue as reporter was also employed in miRNA analysis with a very low LOD of 2.16 fM, 50 aM and 100 fM, respectively [65–67]. Furthermore, microRNA targets were simultaneously captured by AuNPs and hollowed Au/Ag alloy nanocuboids, both conjugated to DNA probes half-complementary to the target, increasing the SERS signal and achieving an LOD of 0.7 fM in solution [68]. A quartz crystal microbalance (QCM) sensor was also reported for the detection of as low as 10 pM of a miRNA sequence. The method was based on signal amplification through DSN, while the detection was performed using gold nano-dendrimers [69]. Another application involved silica-coated gold nanobipyramids along with an absorbed near infrared (NIR) fluorescent dye (Cy7), which fluorescence was quenched by Cu^2+^ ions. MiRNA target initiated an RCA that released pyrophosphate (PPi) during DNA polymerization. PPi were then bound to Cu^2+^ ions leading to fluorescence recovery and “turn on” of the sensor. The method offered an LOD of 8.4 pM (Fig. 1) [70].

Finally, in the presence of miRNA target, AuNPs were linked to magnetic nanoparticles through hybridization with specific oligonucleotides probes conjugated to both nanoparticles. AuNPs were also bound, through hybridization, to polymeric particles suspended in the solution. Upon application of magnetic field, the complex of the three types of nanoparticles was collected to the bottom of the reaction tube, leading to a light transmission (the blurred solution became transparent) visible by naked eye. The LOD of the method was 1.67 pM (75 amol) of miRNA derived from human breast cancer cells [71].

#### Circulating tumor DNA

A PNA probe conjugated to gold nanorods on a SPR sensor was used for detecting of as low as 2 nanograms of synthetic single-stranded ctDNA/mL within 10–15 min [72]. SPR was also used for the detection of a specific sequence of ctDNA based on a peptide nucleic acid (PNA) capture probe coupled to AuNPs. The detection limit of the method was 200 fM of ctDNA [73]. A new dual signal amplification SERS nanosensor based on silica-coated Au nanorods was developed for ctDNA detection, as well. The two new metal-carbonyl (metal-CO) SERS labels used did not interfere with the Raman fingerprint region of the DNA and the biomolecules, that compared to other common SERS labels increased the sensitivity of the method. Despite the dual amplification strategy, the LOD was 57.7 nM [74]. Moreover, a colorimetric determination of ctDNA was reported based on three-way target catalytic hairpin assembly (CHA). After CHA, the DNA fragments produced led to aggregation of gold nanoparticles and a change in the color of the solution from red to blue. This system had an LOD of 7.7 fM, but it has not been applied to real samples [75]. Another colorimetric method involved the aggregation of unlabeled AuNPs, as AuNPs bound to isolated ctDNA from blood samples, resulting in a color change visualized by naked eye [76]. A lateral flow strip test was also developed for the detection of a specific mutation in ctDNA. DNA targets were amplified by allele-specific PCR containing a specific oligonucleotide tail at one end. The amplified products were then applied onto the strip and detected by AuNPs via hybridization to the oligonucleotide tail. The authors were able to detect as low as 0.1 fM of amplified ctDNA [77].

#### Circulating tumor cells

A dual amplification method using RCA and AuNPs was developed for circulating tumor cells (CTCs) detection. RCA produced long ssDNA sequences that were captured by DNA-AuNPs conjugates. The captured AuNPs were then measured by inductively coupled plasma mass spectrometry (ICP-MS) with an LOD of 15 CTCs/mL. The method improved the LOD about 94-fold achieving the lowest LOD of ICP-MS-based methods and the sensitivity about 756-fold when only AuNPs were used as enhancers. However, this method had a long analysis time (> 6 h) [78]. Aptamer-modified gold nanofilms (10–100 nm) were used for the identification of CTCs in blood samples through pulsed laser desorption/ionization mass spectrometry (LDI-MS). The authors could detect as low as 10 cancer cells in blood sample [79]. An aptamer-AuNPs-based strip biosensor was also developed for the visual detection of CTCs. The formation of a red line (positive signal) at the test zone of the strip was induced by another biotinylated aptamer coupled to immobilized streptavidin at the test zone through interaction with CTCs-aptamer-gold nanoparticles complexes (Fig. 1). The authors were able to detect a minimum of 4 × 10^3^ CTCs by naked eye and 800 CTCs using a portable strip reader within 15 min [80].

#### Exosomes

Gold nanoparticles have also been used for the detection of exosomes. A dual-enhancement method was developed using AuNPs conjugated to a specific aptamer. The exosomes were captivated by the AuNPs-aptamer conjugates and a second DNA capture probe immobilized on an SPR sensor. Then, another batch of AuNPs conjugated to a poly(A) probe were captured to the AuNPs-aptamer conjugates for extra signal enhancement, leading to a change in the SPR signal. The sensor had an LOD of 5 × 10^3^ exosomes/mL, that was 20 times lower than using single AuNP signal amplification and 10^4^-fold lower than ELISA [81]. In another application, exosomes were isolated by aptamer-magnetic beads releasing DNA probes that were pre-hybridized to the aptamers. The laterals triggered an HCR by opening a hairpin coupled to AuNPs. Subsequently, HCR produced a structure of a fluorescent DNA dendrimer on the AuNPs’ surface, leading to a dual signal enhancement. Fluorescence was finally measured after centrifugation and collection of AuNPs. The LOD of the method was 1.16 × 10^3^ particles/µL [82]. In another report, aptamers conjugated to AuNPs were bound to exosomal proteins inducing aggregation of AuNPs, a subsequent visual change in the color of the particle solution and a final shift in the UV–VIS absorbance. This method offered good specificity and the potential for rapid differentiation of various exosomal proteins [27]. Raman spectroscopy was also exploited for the detection of exosomes using gold nanorods or nanoparticles coated with Raman probes, targeting exosomal proteins. The advantages of this method lied in simplicity and portability, low cost and high efficiency, offering an LOD of 32–203 exosomes/μL [83, 84]. Moreover, a SPR sensor using bare self-assembly gold nano-islands on the sensor’s surface was used for the detection of exosomes with an LOD of 0.194 μg/mL [85]. Finally, a rapid lateral flow immunoassay was also reported for exosomes detection using specific antibody-labeled AuNPs, offering a high LOD of 8.5 × 10^5^ exosomes/μL [86].

### Carbon-based nanomaterials

Carbon nanomaterials, such as gold nanoparticles, have been recently used as supporting materials for signal enhancement due to their excellent conductivity, high specific surface area-to-volume ratio, good biocompatibility, and size-dependent properties [37]. Various carbon-based nanomaterials have been extensively studied for the development of analytical methods for cancer biomarker analysis, serving as excellent electron transfer materials or fluorescence quenching media. Carbon-based materials have various reactive groups on their surface, such as -COOH and -OH, which make them ideal for conjugation to biomolecules. Carbon quantum dots (CQDs) are produced with low-cost and easy fabrication techniques, while they have demonstrated good biocompatibility, low toxicity, strong quantum size effect, excellent electron transfer properties, narrow emission peaks, photostability and resistance to photobleaching [7]. Single-walled carbon nanotubes (SWCNTs) do not photobleach, while their fluorescence in the near-infrared spectral region, which can penetrate tissues, enables bioimaging [87]. They also have catalytic properties, such as peroxidase-like activity, leading to colorimetric sensors development [88]. Moreover, carbon nitride nanosheets exhibit strong fluorescence quenching, low toxicity and good biocompatibility [89]. The carbon-based nanomaterials used in liquid biopsy applications include graphene oxide, carbon nanodots and carbon quantum dots, carbon nanotubes, fullerenes, carbon nitride nanosheets and molybdenum carbide (Mo_2_C) nanotubes.

#### Graphene oxide (GO)

Two-dimensional graphene oxide (GO) is considered as a very attractive carbon-based nanomaterial with numerous applications in biosensing, bioimaging, drug delivery and energy storage. Several electrochemical and spectrometric biosensors have been developed for biosensing in recent years [90]. GO provides a large surface area serving as a signal-enhancement new platform, exhibits excellent electronic/electrical properties, and provides various functional groups and multiple active sites for bioconjugations. For fluorescent applications, GO has attracted great attention due to its adsorption capacity for ssDNA and its superior quenching ability [91]. GO has been used for the development of electrochemical and fluorometric sensors, as well as for homogeneous assays with very low LODs and applications to liquid biopsy samples.

##### MicroRNAs

**Fig. 2:**
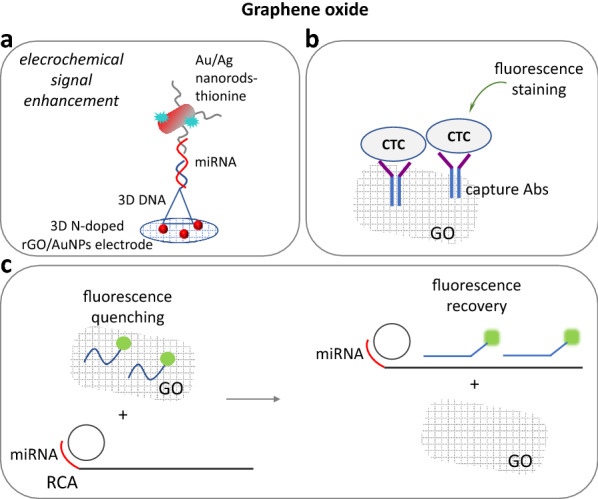
**a** Electrochemical detection of miRNA based on gold and silver nanorods and the reductive compound thionine. The overall structure is captured on the electrode’s surface via a nitrogen-doped reduced GO/AuNPs/tetrahedral DNA nanostructure. **b** An antibody-reduced GO for CTCs sensing. **c** A fluorescent biosensor based on fluorescence quenching/recovery by GO using fluorescent DNA probes and rolling circle amplification (RCA) triggered by miRNA target. *GO* graphene oxide, *CTCs* circulating tumor cells, *AuNPs* gold nanoparticles

Graphene oxide was also used for miRNA detection. A GO-fluorescence based assay that involved ssDNA and RCA was developed for miRNA analysis with an LOD of 0.87 fM [92]. Using different fluorescent ssDNA, RCA and GO for fluorescence quenching, Treerattrakoon et al. (2019) developed a multiplex miRNA sensing system with an LOD of 0.05 pmol (Fig. 2) [93]. HCR-induced signal amplification was exploited by Fan et al. (2018) and Zhen et al. (2017) for miRNA detection, using GO and fluorescence recovery phenomena. The authors achieved a low LOD of 4.2 fM and 47 pM, respectively [94, 95]. The same concept was applied for miRNA imaging in living cells (5.5 × 10^3^ copies/cell) [96]. Moreover, an HCR was triggered by an RNA target after hybridization to a capture probe immobilized on Ru(phen)_3_^2+^/Fe_3_O_4_-SiO_2_/AuNPs nanoparticles. The HCR products were then captured through hybridization on the GO’s surface leading to fluorescence quenching [97]. In another report, ssDNA sequences were covalently coupled to GO’s surface. The authors used fluorescently labeled DNA probes that contained two segments: one part complementary to the immobilized DNA sequence and one part complementary to a miRNA sequence. The DNA probes came to close proximity to the GO through hybridization to the ssDNA sequences on the GO’s surface, leading to fluorescence quenching. Upon addition of miRNA target, fluorescence was recovered. The method had an LOD of 10 pM and 181 pM, respectively [18, 98]. Furthermore, a helicase-based hybridization reaction on GO using a fluorescently-labelled DNA probe offered a detection limit of 180 pM [99]. Robertson et al. (2017) achieved to detect a specific miRNA molecule, among two other miRNAs, using 2D-GO and fluorescently labeled unlocked DNA specific probes. The authors proved that the addition of the specific endonuclease dsDNase resulted in signal enhancement, but the method was not tested in real samples [100]. A novel method for miRNA detection was also developed by Esteban-Fernández de Ávila et al. (2015) based on dye-labeled ssDNA/graphene-oxide complex coated with gold nanowires that were capable of penetrating cancer cells, while Ryoo et al. (2013) used fluorescent Peptide Nucleic Acid (PNA) probe and GO for the same aim with an LOD of 1 pM. The fluorescence signal was recovered upon binding of the fluorescent ssDNA or PNA probe to the target miRNA [101, 102]. A signal enhancement was also achieved using exonuclease III that digested the hybrids between a DNA probe and target miRNA, producing small oligonucleotides that significantly increased the fluorescence of rhodamine 6G (R6G) dye. More specifically, the dye molecules were displaced from the R6G-GO complex by the oligonucleotides leading to fluorescence recovery. The method offered a low detection limit of 1.0 fM [103]. A colorimetric detection of miRNA was also developed using a duplex molecular beacon. The hybridization of target miRNA was accomplished through strand displacement inducing the release of peroxidase-mimicking DNAzyme which was gathered onto GO. Finally, the DNAzyme catalyzed a hemin-based colorimetric reaction. The method had an LOD of 12.9 nM [104]. Another electrochemical system involved the immobilization of a DNA probe on an electrode. Target miRNA was then hybridized to the specific probe, while GO was aggregated on the hybrids, accumulating methylene blue dye that reduced the pulse voltammetric signal. DSN was then used for target recycling and signal amplification, offering an LOD of 10 aM [105]. A similar approach introduced the *in-situ* formation of Prussian Blue nanoparticles on GO’s surface for the electrochemical sensing of as low as 1.5 fM of miRNA [90].

The combination of GO with other nanoparticles set the basis for a sensitive detection of biomolecules. Gold nanoparticles and other metal particles coupled to GO accelerate the electron transfer, that is exploited for the development of electrochemical and optical sensors [106]. Nitrogen-doped reduced GO with Au/Ag nanorods coupled to a tetrahedral DNA nanostructure was constructed on an electrode’s surface for electrochemical sensing of as low as 1 pM of miRNA. The method was based on a gold and silver nanorod/thionine/complementary DNA probe assembly that used to capture the miRNA target, leading to thionine reduction on the electrode’s surface (Fig. 2). Compared to common ssDNA/RNA probes, the DNA tetrahedral nanostructure eliminated steric hindrance effects, providing a solution-like environment, increasing the target accessibility and enhancing the sensor’s performance [33]. Again, an interesting system including AuNPs/polypyrrole-reduced GO to provide a large surface area and a high-conductive platform, combined with CHA and HCR was constructed for multiple signal amplification and electrochemical detection of miRNA. Methylene blue was used as the signal indicator and the method offered an LOD of 1.57 fM [37]. A novel electrochemical biosensor was based on multi-walled carbon nanotubes (MWCNTs)/AuNPs/GO nanoribbons and DSN-assisted target recycling in combination with the reduction of ascorbic acid by alkaline phosphatase for the analysis of miRNA. The method achieved an LOD of 34 aM [107]. Another application involved GO decorated with gold-platinum bimetallic nanoparticles that was formed onto fluorine tin oxide sheets and used for the voltammetric detection of miRNA through a captured DNA probe. The sensor provided an LOD of 1 fM and also a 3-times re-usability [108]. A further miRNA electrochemical sensing system involved the hybridization of target miRNA onto DNA-conjugated AuNPs. After hybridization, DSN hydrolyzed the DNA probe from the duplex, releasing the target for target recycling and exposing the surface of AuNPs. AuNPs were finally captured by GO on an electrode following electrocatalytic signal amplification. The method demonstrated an LOD of 1.5 fM [109]. GO was also modified with gold nanoparticles offering an LOD of 0.1 fM—1.74 nM of miRNA [106, 110, 111], and with magnetic silicon microspheres with an LOD of 98 pM [91]. An assembly of ZrO_2_-reduced GO nanohybrids coupled to CHA-based signal amplification reaction was also developed for a label-free impedimetric sensing of miRNA molecules with an LOD of 4.3 fM [112]. A multifunctional nanocomposite of poly (L-lactide) and polyethylene glycol-grafted GQDs was reported for simultaneous intracellular miRNAs imaging analysis that showed stable photoluminescence over a broad pH range, which is vital for cell imaging [113]. Next, a complex of GO with QDs and HCR (fluorescence switch “on”- “off”) was used for the detection of as low as 102 tumor cells or 1 pM miRNA [114]. GO was also modified with disposable graphite electrodes for the electrochemical detection of miRNA through amino-linked miRNA-specific DNA probes, offering an LOD of 702.7 pM [115].

##### Circulating tumor cells

An antibody-modified reduced GO was constructed for CTCs sensing. The captured cells were detected by immunostaining method, achieving an LOD of 2 CTCs/4 mL blood (Fig. 2) [116]. An aptasensor using tetra(4-aminophenyl) porphyrin mediated reduced GO was constructed for the electrochemical detection of as low as 10 CTCs/mL [117]. Another aptasensor was developed based on hairpin aptamer probes, dye-labeled linker DNA probes, nicking endonuclease and GO for the detection of 25 cancer cells in a blood sample [118]. Again, the modification of GO with AuNPs was used for the electrochemical detection of as low as 40 CTCs/mL, based on a ferrocene-aptamers/Ru(bpy)_3_^2+^/β-cyclodextrin-AuNPs/GO complex, while this aptasensor could be re-used for 6 more cycles [119].

##### Exosomes

Exosomes have been detected using DNase I enzyme-aided fluorescence signal amplification based on GO-DNA aptamer interactions achieving a detection limit of 2.1 × 10^4^ particles/μL of colorectal cancer exosomes. A fluorescently labeled DNA aptamer was firstly absorbed onto GO followed by fluorescence quenching. In the presence of exosomes, the DNA aptamer was released from GO due to the strong affinity to the exosomes with subsequent fluorescence recovery. Treatment with DNase I digested the ssDNA aptamers that released the exosomes being available to interact with new aptamers, and led to fluorescence signal enhancement [120]. Moreover, the peroxidase-like activity of graphitic carbon nitride nanosheets in combination with ssDNA probes was exploited for the colorimetric detection of as low as 13.5 × 10^5^ exosomes/μL [121]. A microfluidic exosome immune-analysis platform based on a new graphene oxide/polydopamine (GO/PDA) nano-interface was developed followed by a sandwich-type ELISA for exosomes detection. The method was based on enzymatic fluorescence signal amplification, and achieved a very low detection limit of 50 exosomes/μL [122].

#### Other carbon nanoparticles

**Fig. 3:**
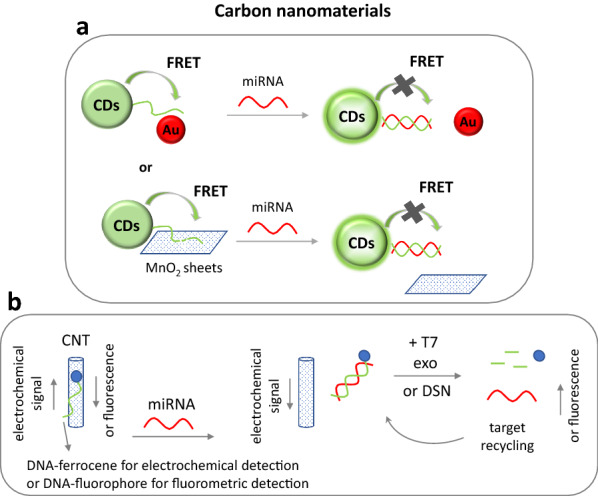
**a** Fluorescent carbon dots (CDs) coupled to a DNA probe are quenched by gold nanoparticles (Au) or MnO_2_ nanosheets, phenomenon that is then hindered by hybridization of miRNA target. **b** DNA-carbon nanotubes with T7 exonuclease-assisted or DSN-assisted target recycling were used for the electrochemical or fluorometric detection of miRNA. *CNT* carbon nanotube, *exo* exonuclease, *FRET* fluorescence energy transfer, *DSN* duplex-specific nuclease

Several carbon nanomaterials in combination with other materials have been exploited for optical and electrochemical sensing with applications to liquid biopsy. More specifically, a combination of carbon nanotubes and lysozyme-modified gold nanoclusters with fluorescently labeled ssDNA was introduced for the detection of specific miRNA sequences. The hybridization of ssDNA to target miRNA led to fluorescence recovery with an LOD of 36 pM [123]. Also, multi-walled carbon nanotubes (MWCNTs)-gold nanocomposites combined with DSN target recycling strategy were used as a novel fluorescence quenching platform for ultrasensitive detection of miRNA with an LOD of 33.4 fM (Fig. 3) [8]. DNA-coupled to carbon nanotubes onto an electrode, exploiting T7 exonuclease-assisted target recycling, were also used for the electrochemical detection of 3.5 fM of miRNA (Fig. 3) [11], while single-walled carbon nanotubes (SWCNTs) coupled to silver nanoparticles and a complex of AuNPs/carbon spheres-MoS_2_ combined with HCR were exploited for electrochemical miRNA detection with an LOD of 313 fM and 16 aM, respectively [124, 125]. Electrochemical miRNA sensing was also performed using nitrogen-doped hollow carbon nanospheres (LOD 0.1 fM) [126], carbon black nanoparticles (LOD 10 pM) [127], carbon nanofibers with an LOD of 1.54 μM [128] and a combination of oxidized SWCNTs, nanodiamonds, AuNPs and HCR with an LOD of 1.95 fM [129]. A novel photoelectrochemical biosensor was developed for dual sensitive detection of microRNAs using molybdenum carbide (Mo_2_C) nanotubes as nanocarriers and energy transfer between carbon quantum dots (CQDs) and AuNPs. The CQDs@Mo_2_C were deposited onto an ITO electrode, while two hairpin probes carrying the AuNPs were used for signal “switch off” and “switch on”, when AuNPs were in close proximity to the CQDs quenching the photoelectrochemical signal. The addition of different targets altered the distance between the particles, changing the signal response. The sensor had an LOD of 0.15 fM [130]. A photoelectrochemical biosensor was also constructed using a fullerene/poly(ethylene glycol) nanocapsule that contained a donor–acceptor-type photoactive complex for the determination of as low as 83 aM of miRNA [131], while MWCNTs were also introduced for the immuno-electrochemical detection of prostate-specific antigen with an LOD of 20 pg/mL [132].

A ratiometric sensing system was developed using fluorescent carbon dots coupled to miRNA probe and rhodamine dye which fluorescence was quenched due to fluorescence energy transfer (FRET) mechanism by AuNPs, a phenomenon that was hindered upon target hybridization. The method had a very low LOD of 0.3 aM (Fig. 3) [133]. Fluorometric determination of miRNAs was also achieved using carbon dots in combination with molecular beacon and fluorescence enhancement upon target hybridization (LOD 0.3 nM) [7], MnO_2_ nanosheets (LOD 0.1 aM) (Fig. 3) [134], carbon nitride nanosheets combined with HCR (LOD 0.10 pM) and application in living cells [80], or SWCNTs combined with SDA (LOD 10–100 pM) [87].

SWCNTs and SERS signaling were used for the detection of ctDNA offering an LOD of 0.3 fM [135]. Finally, a colorimetric aptasensor based on DNA-capped (aptamer) SWCNTs was developed, exploiting the peroxide-like activity of carbon nanotubes, for the detection of exosomes with an LOD of 5.2 × 10^5^ particles/μL. The LOD was 10 times lower than the common immunoassay that used specific antibodies for the detection [88]. Finally, carbon nitride nanosheets decorated with AuNPs were used for the electrochemiluminescent detection of only 2 CTCs in a sample [9].

### Quantum dots (QDs)

QDs have also played a key role in novel methods for liquid biopsy applications. QDs are popular due to their unique optical properties (i.e., their wide excitation spectra and narrow size-depended emission peaks, bright fluorescence and great photostability) compared to organic fluorescent dyes, which make them ideal in biosensing with high sensitivity and high multiplex capability [6]. Scientists have also combined QDs with several other nanomaterials, either in order to increase the analytical performance of the methods as a result of the synergetic effects in signal improvement or, in some cases, to improve their biocompatibility, using more compatible to human blood/serum samples nanomaterials.

A great number of QDs-derived methods have been developed so far for miRNA detection. These methods are mostly based on lateral flow assays, signal “on”- signal “off”, FRET and electrochemiluminescence [6]. A novel electrochemiluminescent biosensor was constructed for miRNA detection. The sensor was based on a Zn^2+^−driven DNA rolling machine and target recycling for signal amplification. The DNA nanomachine was constructed by AuNPs coupled to specific DNA probes that were hybridized and walked across attached complementary DNA probes with Zn^2+^ recognitions sites on an electrode’s surface. CdS@Mn QDs were also employed on the electrode’s surface as the luminescent substrate for the electrochemiluminescent signal, along with ferrocene for signal quenching. Ferrocene was then removed by Zn^2+^-driven cleavage and the rolling of the DNA nanomachine after capturing, restored the electrochemiluminescent signal. The target-induced recycling reaction resulted in a large amount of Zn^2+^, improving the detectability of the biosensor, achieving an LOD of 0.28 fM [136]. A new photoelectrochemical biosensor based on a photocurrent direction switching system and target-triggered SDA strategy was developed for miRNA detection with an excellent detection limit of about 49 aM [137]. A novel photoelectrochemical sensor for miRNA detection was also reported based on energy transfer between CdS:Mn dots and AuNPs, succeeding an LOD of 0.5 fM [138]. Another aptasensor based on CdSe QDs and HCR offered an LOD of 5.6 fM [139]. Furthermore, a DNA tetrahedron as nanocarrier was constructed for efficient immobilization of CdTe QDs for miRNA photoelectrochemical detection based on QDs-methylene blue complex and enzyme-assisted target cycling amplification, achieving a very low LOD of 17 aM [140]. A dual channel ratiometric nanoprobe was described for the detection and imaging of miRNA based on MoS_2_-QDs and a molecular beacon that carried the fluorescent dye fluorescein at one end. Molecular beacon was coupled to the QDs leading to low FRET efficiency. Upon addition of miRNA that hybridized to the molecular beacon, the distance between the fluorescein and the QDs increased to the optimum, resulting to stronger FRET efficiency fluorescence increasement. This system offered an LOD of 0.52 nM [141].

**Fig. 4:**
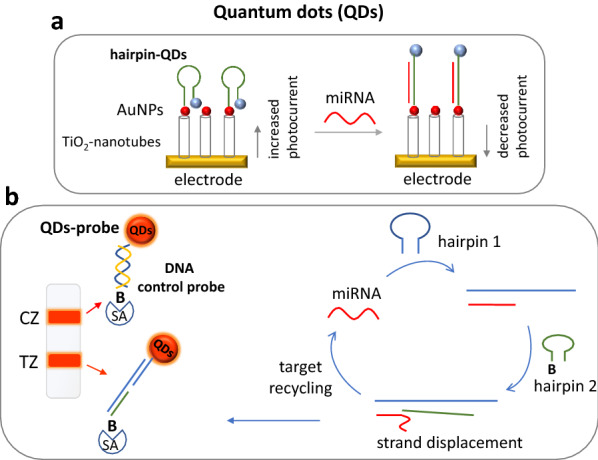
**a** A photoelectrochemical sensing system based on a decrease in the signal, as quantum dots (QDs) are moving away from the electrode’s surface upon hybridization of target miRNA to DNA hairpin/AuNPs/TiO_2_NPs-decorated fluorine-tin oxide electrodes. **b** A lateral flow assay based on QDs-DNA as reporters and SDA target amplification for miRNA detection. *AuNPs* gold nanoparticles, *TZ* test zone, *CZ* control zone, *SA* streptavidin, *B* biotin

The first lateral flow assay based on QDs-DNA as reporters and SDA target amplification for miRNA detection was reported by Deng et al., with an LOD of 200 amol (10 pM) of miRNA. The detectability was tenfold lower than using a conventional gold nanoparticle-based strip (Fig. 4) [142].

CdTe QDs incorporated into mesoporous silica nanoparticles revealed stronger electrochemiluminescent signal than single QDs and were used for miRNA detection. These QDs were combined with DSN-assisted target amplification approach on Fe_3_O_4_@Au nanoparticles, achieving an LOD of 33 fM [143]. A dual miRNA detection was reported by a photoelectrochemical biosensor using two different CdTe QDs. The QDs were loaded onto carbon nitrides nanosheets with anodic photocurrent and on a 3D graphene hydrogel with cathodic photocurrent. Two different DNA probes specific to two miRNAs were covalently coupled to the two nanoplatforms, respectively. The hybridization of target miRNA to the complementary DNA was monitored by the respective photocurrent change achieving an LOD of 1 fM [144]. Moreover, a QD-molecular beacon platform was tested for monitoring and imaging of intracellular miRNAs [145]. The energy transfer between CdS QDs and oligonucleotide encapsulated silver nanoclusters was also exploited for the electrochemiluminescent detection of as low as 10 fM of miRNA [146].

The combination of MoS_2_-QDs with GO and AgNCs-polyamidoamine was also reported for the electrochemiluminescent miRNA detection with an LOD of 0.2 fM [147]. The energy transfer between CdTe QDs and silver nanoclusters was also exploited for miRNA detection with an LOD of 1.2 pM [148]. Moreover, a dual-target HCR and exonuclease III-aided target recycling process was reported for miRNA detection with an LOD of 1.5 pM [149] and a fluorescent QDs-liquid bead array using specific captured DNA probes and quantitative reverse transcriptase polymerase chain reaction was successfully constructed for the simultaneous detection of 12 different miRNA molecules [150].

An aptamer-based graphene QDs/Fe_3_O_4_ assembly in combination with molybdenumdisulfide (MoS_2_) nanosheets as fluorescence quencher were used for the enrichment and detection of as low as 10 CTCs/blood sample [151]. Also, QDs in combination with magnetic nanoparticles was used for the isolation of CTCs captured by immobilized aptamers. The isolation was completed within 20 min achieving single cell detection [152]. Moreover, RCA combined with QDs and stripping voltammetry producing electrochemical signals of Cd^2+^ was also used for cancer cells detection down to 10 cells/mL [153].

A bead-based microarray was constructed for multiplex exosomes detection using captured antibodies and QD-labeled secondary antibodies [154]. Finally, CdSe QDs were also used for the detection of exosomes via captured specific antibodies and stripping voltammetric quantification of Cd^2+^. This method offered the detection of as low as 100 exosomes/μL [155].

### Copper nanoparticles (CuNPs)

Metallic nanoclusters have a small size (~ 2 nm), high conductivity and a large surface area, intrinsic fluorescence and quantum effects, chirality and ferromagnetism properties which are sometimes not present even in nanoparticles [44]. Several metallic nanostructures have exhibited peroxide-like activity, which has been exploited for the oxidation of chromogenic substrates, in the presence of hydrogen peroxide, usually in smaller amounts than the enzymes. Copper is usually preferred over other metals due to its low price.

**Fig. 5:**
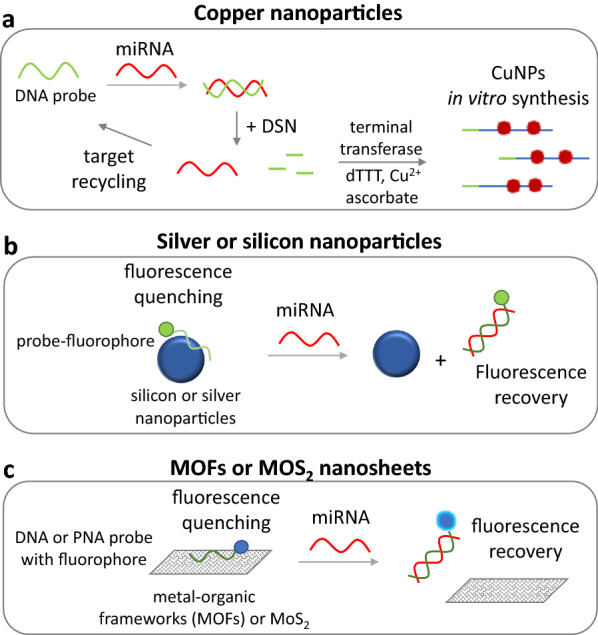
**a** A fluorescent detection of miRNA based on the in vitro synthesis of copper nanoparticles (CuNPs) by terminal transferase and ascorbate oxidation. The signal is enhanced by DSN-assisted target recycling. **b** A fluorescent detection of miRNA based on silicon or silver nanoparticles. The fluorescence of a single-stranded DNA probe is quenched by the nanoparticles, while fluorescence is recovered by target miRNA hybridization to the probe. **c** In this approach, the fluorescence of a single-stranded DNA probe is quenched by metal–organic frameworks (MOFs) or MoS_2_ nanosheets, which is then recovered by miRNA target hybridization to the DNA probe. *DSN* duplex-specific nuclease

Visual detection of miRNA was achieved by peroxidase-like catalytic activity of DNA-Cu nanoclusters and methylene blue as indicator. The method offered an LOD of 0.6 pM [156]. Compared to other fluorescent nanoparticles, copper nanoparticles and nanoclusters can be synthesized in situ through a simple reduction of Cu^2+^ by ascorbate on DNA scaffolds (dsDNA or polyT/AT ssDNA) with the following advantages: (i) the synthesis is rapid and can be completed within 10 min, (ii) Cu is biocompatible and (iii) CuNPs emit strong red fluorescence, which is well distinguished in biological systems. So, a novel method has been developed by Xu et al. (2018) that used DSN which integrated target amplification and resulted in terminal deoxynucleotidyl transferase-mediated polyT-CuNPs synthesis for fluorescent detection of miRNA molecules. The method had an LOD of 20 pM (Fig. 5a) [157]. The in situ synthesis of copper nanoblocks (CuNBs) coupled to a DNA probe and with subsequent hybridization-based magnetic isolation was used for the detection of 500 fM of miRNA in solution based on fluorescent readout and 100 fM after electrochemical analysis [158]. Target-driven CuNPs synthesis on a DNA tetrahedron was also utilized for electrochemiluminescent miRNA detection (LOD = 36 aM) [159]. In another report, target miRNA initiated an HCR upon hybridization to a hairpin bound to an electrode. The HCR with the assistance of exonuclease III and TiO_2_/Pt NPs produced AT-rich dsDNA, where Cu nanoclusters were formed. An electrochemiluminescent signal was then generated and the method had an LOD of 19.05 aM [160]. Borghei et al. (2017) took advantage of the shift in the fluorescence of a DNA-Cu nanoclusters complex after hybridization to the miRNA target, detecting as low as 2.2 pM of target miRNA [161]. Similarly, the *in-situ* synthesis of Cu nanoclusters after the hybridization of target miRNA to a Y-shaped DNA probe on an electrode’s surface, followed by exonuclease T7 target recycling, strand displacement and HCR, offered an extremely low LOD of 10 aM [162]. Again, poly-thimine sequences were incorporated via terminal deoxynucleotidyl transferase into target miRNA which served as the template for in situ formation of fluorescent CuNPs for detecting miRNA with an LOD of 100 fM [163].

Finally, aptamer-modified copper oxide nanoparticles (CuONPs) were used for exosomes capturing. The CuONPs were turned to fluorescent CuNPs upon addition of acid, sodium ascorbate and poly-thymine bases, offering an LOD of 4.8 × 10^4^ exosomes/μL [30].

### Silver nanoparticles (AgNPs)

Silver nanoparticles have been widely used as signal enhancers, especially in electrochemical, SPR and SERS applications, as they are easily oxidized and reveal higher extinction coefficients than AuNPs [4]. Molecules of miRNA were detected by CHA using two hairpins: one hairpin contained silver nanoclusters (AgNCs), while the other contained G-rich DNA sequences. The hybridization of miRNA to the first hairpin triggered CHA, while AgNCs came in close proximity with the G-rich regions that significantly enhanced the fluorescence of AgNCs. The detection limit of this method was 0.3 nM [164]. Another report was based on a dual, colorimetric and fluorometric, sensing system that involved the quenching of AgNCs coupled to DNA probes by AuNPs, when the DNA probe interacted with the AuNPs. Upon addition of the target, miRNA was hybridized to the DNA probe releasing the AgNCs from the AuNPs, resulting in fluorescence recovery and in salt-induced aggregation of AuNPs that changed the color of the solution from red to purple. The method offered an LOD of 0.6 nM for the colorimetric and 0.4 pM for the fluorometric approach (Fig. 5b). Silver nanoclusters is a promising label, as they show high fluorescence quantum yield, photostability and excellent biocompatibility [165]. A similar strategy was developed using AgNCs coupled to a DNA probe that was hybridized to target miRNA. Positively charged AuNPs were adsorbed to the negatively charged DNA probe quenching the fluorescence of AgNCs. By enzymatic hydrolysis of the DNA-RNA hybrid by DSN, the AuNPs were removed, resulting in fluorescence recovery. This system offered an LOD of 33.4 fM [166]. Moreover, a novel Au/Ag nanocube coupled to a tetrahedron DNA structure was constructed for the detection of microRNA at the single-molecule level with an extremely low LOD < 1 aM. The detection was based on surface plasmon resonance scattering spectral wavelength shift upon hybridization of target miRNA [167]. In situ formation of silver nanoparticles was also introduced for signal amplification and miRNA electrochemical detection with an LOD of 20 aM [168]. Moreover, origami paper analytical devices modified with DNA-encoded Raman-active anisotropic silver nanoparticles were designed for miRNA detection (LOD 1 pM) [169].

A SERS system on a silver nanorods array combined with a primary target-triggered enzyme-free recycling and a secondary signal enhancement step using multiple reporters was developed for the detection of as low as 40.4 aM of ctDNA [170].

Silver nanoprisms in combination with magnetic iron oxide nanoparticles were applied for the sensitive detection of CTCs via magnetic enrichment and SERS detection system, offering a very low LOD of 1 cell/mL [171], while aptamer-modified Ag/Au core–shell nanoparticles were used for CTCs detection by localized surface plasmon resonance (LSPR) with an LOD of 10 cells/mL [172].

Polydopamine-encapsulated antibody-reporter-Ag(shell)-Au(core) nanoparticles were prepared as SERS probes for exosomes detection with a detection limit of only one exosome in 2 μL of sample solution [173]. Finally, AgNPs conjugated with peptide ligand were used to capture exosomes, while the detection was accomplished through SERS [174].

### Silica nanoparticles (SiNPs)

Silica or silicon nanoparticles have been exploited in many different analytical detection strategies and in drug delivery, because various molecules can be physically or chemically encapsulated in a single silica nanoparticle. Dye molecules or electroactive molecules can also be encapsulated in SiNPs, increasing detectability, instead of the dye itself being used as a common label [34]. In addition, silica nanomaterials can be synthesized by means of a cheap and facile hydrothermal method, as they exhibit strong fluorescence, great photostability, good water solubility, long lifetime and low toxicity [175].

SiNPs were also used in miRNA sensing. More specifically, in one approach, a Cy5-labeled DNA probe was linked to SiNPs that quenched the fluorescence of Cy5 which was then restored upon hybridization of target miRNA. The system demonstrated an LOD of 0.16 nM (Fig. 5b) [175]. In another approach, the target miRNA triggered HCR and CHA using two DNA hairpins that contained G-quadruplex DNA sequences, leading to the formation of a horseradish peroxidase-mimicking DNAzyme. This DNAzyme catalyzed the oxidation of o-phenylenediamine to the fluorescent product 2,3-diaminophenazine in the presence of H_2_O_2_. The fluorescence of the product was then quenched by SiNPs. Even though three different amplification systems were used here, the method did not offer a good detectability and had an LOD of 2.5 pM. In contrary, the method had great selectivity even in 1-base mismatched targets [176]. SiO_2_ nanofibres surrounded by upconversion luminescent nanoparticles were synthesized for miRNA detection. The method was based on hybridization of target miRNA to an immobilized to the sensor’s surface molecular beacon. The molecular beacon was then coupled to a quencher molecule, leading to luminescence recovery. This sensor offered a quite high LOD of 2 nM [177]. Another approach used DNA-SiNPs conjugates for miRNA detection. The immobilized DNA probe carried a fluorophore (fluorescein). A second smaller probe that carried a quencher molecule at one end was hybridized to the immobilized probe leading to fluorescence quenching. The target miRNA could displace the quencher DNA-probe restoring the fluorescence [178]. Finally, polyethyleneimine SiNPs modified with Cu^2+^ were used as an electrochemical sensor for miRNA detection based on hybridization of target miRNA with the complementary DNA probe. The LOD of the method was 30 fM [34].

### Iron, magnetic and superparamagnetic nanoparticles

Magnetic nanoparticles have been frequently utilized in biosensing methods, mainly due to signal enhancement and simplicity of the isolation and washing-step procedure, to remove unbound reagents and to provide analyte enrichment, minimizing the complexity of human biological samples and increasing the detectability and specificity of the methods. Some magnetic nanomaterials have also shown peroxidase-like activity, which has led to the development of colorimetric sensing systems [4]. Magnetic nanomaterials have been utilized for analytical sensing applications due to their flexible and modular structure, easy synthesis, low toxicity, high biocompatibility, enzyme-mimicking activity, superparamagnetic behavior and bioconjugation to a wide range of biomolecules. Their simple and rapid isolation from the solution in the presence of a magnetic field makes them ideal as alternative solid support for the development of highly specific and sensitive analytical assays. Because they provide isolation, purification and target-molecule capturing, magnetic materials have been also involved in signal-enhancement steps in biosensing [35].

Gold-coated paramagnetic nanoparticles have recently used to enhance the SERS signal. The gold-decorated paramagnetic nanoparticles were coupled together with Raman-tagged AuNPs or silica-coated AuNPs through conjugated DNA probes that were subsequently hybridized to the target miRNA. The method had an LOD of 100 fM or 1.8 fM, respectively, which improved the LOD by a 100-fold compared to non-plasmonic metal nanoparticles [179, 180]. Gold-loaded nanoporous superparamagnetic iron oxide nanocubes were also used for the electrocatalytic detection of miRNA using two reducing agents, offering a low LOD of 100 aM [181]. Furthermore, gold-coated magnetic nanoparticles attached to a DNA probe were used for the detection of as low as 10 aM of miRNA in buffer solution, which is approximately 10,000,000 times lower than using the same detection strategy on a planar surface. The DNA probe was labeled with methylene blue and was complementary to the target miRNA. The detection of the formed hybrids was accomplished through square-wave voltammetry [182]. Graphene-oxide-loaded superparamagnetic iron oxide nanoparticles have also been used for the electrocatalytic detection of as low as 1 fM of target microRNA. The detection was based on the reduction of the [Ru(NH_3_)_6_]^3+^/[Fe(CN)_6_]^3+^ system [183].

**Fig. 6:**
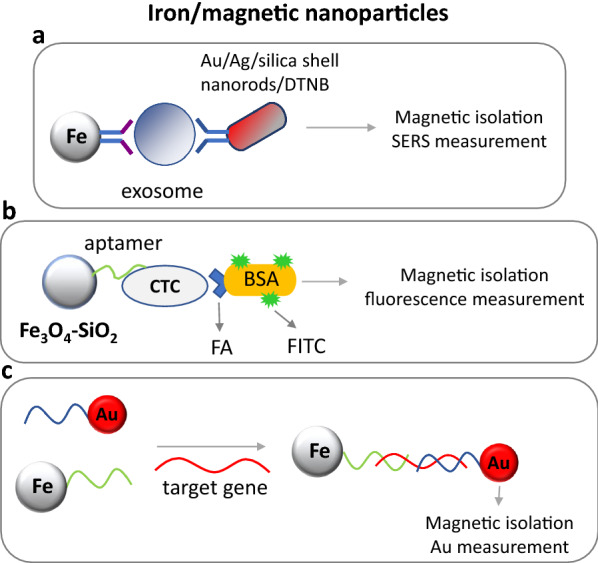
**a** Exosomes detection using Au/Ag/silica shell nanorods and magnetic nanoparticles coupled to specific antibodies and SERS measurement. **b** Circulating cancers cells (CTCs) are detected by magnetic Fe_3_O_4_-silicon nanoparticles conjugated to a specific aptamer. The detection was achieved by FITC/FA-labeled BSA. **c** MiRNA is detected by hybridization to both DNA-conjugated gold and magnetic nanoparticles, while Au concentration is measured by inductively coupled plasma mass spectrometry, after magnetic isolation. *Fe* magnetic nanoparticles, *Au* gold nanoparticles, *FITC* fluorescein, *FA* folic acid

Moreover, magnetic amorphous Fe@SiO_2_ nanoparticles conjugated to a DNA probe 1 and AuNPs coupled to a DNA probe 2 were used for the detection of ctDNA. The silica coating protected magnetic nanoparticles form air oxidation and increased their solubility in water, and their biocompatibility, while it enabled bioconjugation. Upon target hybridization, both nanoparticles came to close proximity and were subsequently magnetically isolated. Then, the Au concentration was measured by inductively coupled plasma mass spectrometry (ICP-MS), detecting as low as 0.1 pg/mL ctDNA (Fig. 6) [184]. Streptavidin-paramagnetic iron oxide particles were also used for the detection of as low as 50 copies of ctDNA using a coated microfluidic biochip [185].

The approach that gave one of the best detectabilities among the reported values for CTCs detection was based on SERS-active magnetic nanoparticles. More specifically, poly(ethyleneimine)-stabilized superparamagnetic iron oxide nanoparticles in combination with AuNPs were used for the sensitive detection of as low as 1 CTC/mL based on SERS signal [186]. Magnetic Fe_3_O_4_ nanoparticles in combination with SiNPs conjugated to anti-MUC1 aptamer were also used for the detection of cancer cells. The authors were able to detect 100 cells/mL (Fig. 6) [187]. A 3D matrix synthesized by crosslinking polyethylene glycol-Fe_3_O_4_ nanostructures was finally used in continuous flow microchannel for the isolation and fluorescent detection of CTCs. The matrix was linked to the target ligand Tf, while down to 25 cells/mL were captured by this system [188].

Superparamagnetic iron oxide nanoparticles were also used for the detection and imaging of exosomes. The detection was accomplished via magnetic resonance imaging and the LOD was 100 and 2.5 × 10^3^ cells in vitro and in vivo, respectively [189]. Also, Fe_3_O_4_ magnetic nanoparticles in combination with Au–Ag nanorods as SERS probes were used for the detection of exosomes. The magnetic nanoparticles were covered with a silica shell on which specific antibodies were attached. Exosomes were then detected by a sandwich-type immunoassay using a second specific antibody coupled to the Au–Ag nanorods generating a SERS signal. This sensor gave an LOD of 1200 exosomes, approximately (Fig. 6) [190]. Again, gold-loaded nanoporous superparamagnetic iron oxide nanocubes coupled with an exosome-specific antibody provided an LOD of 10^3^ exosomes/mL [191]. Finally, streptavidin-magnetic nanoparticles were interrogated in an SPR sensor for the detection of extracellular vesicles (EVs) using a biotinylated ligand and a specific antibody bound to the sensor’s surface [192].

### Molybdenum disulfide (MoS_2_) nanostructures

Novel molybdenum disulfide nanostructures have been exploited in liquid biopsy testing. Nanostructured MoS_2_ particles are transition metal dichalcogenides and have been an attractive label due to their outstanding thermal properties and metal-like electrical conductivity. They require an easy and low-cost preparation process: they can be easily exfoliated to very few layers, or even a single layer, due to the weak van der Waals interactions in their structure. They also possess high stability and excellent electrical conductivity, while they can react with numerous other nanomaterials forming new novel nanocomposites [130, 193]. MoS_2_ nanosheets have been used for signal enhancement by increasing the surface area and also improving the electron transfer properties. In addition, MoS_2_ nanostructure has similar properties and similar quenching effects to graphene-like nanomaterials and can be applied to fluorometric DNA sensing applications. A recent method involved MoS_2_/Ti_3_C_2_ nanohybrids in combination with AuNPs deposited on a glassy carbon electrode. An RNA probe was then captured on the electrode’s surface through Au–S bond, while the hybridization of target miRNA was recorded by differential pulse voltammetry. The method gave an LOD of 0.43 fM [194]. A novel MoS_2_ 2D nanomaterial was constructed, exhibiting a similar performance to GO in biosensor development. A fluorescently labeled ssDNA probe was absorbed onto the MoS_2_ nanomaterial or folic acid-polyethylene glycol-functionalized MoS_2_ nanosheets, inducing fluorescence quenching. Upon addition of complementary target miRNA, the hybrid was released from the nanomaterial and fluorescence was recovered. The sensors offered a detection limit of 500 pM within 40 min (Fig. 5c) and < 10 nM, respectively [195, 196]. A similar approach used 2D MoS_2_ nanomaterial and an RNA probe for electrochemical detection of miRNA with an LOD of 0.03 fM [197]. Moreover, MoS_2_ nanosheets in combination with molecular beacons for fluorescence quenching and DSN for fluorescence recovery and signal amplification lowered the detection limit of miRNA by about 4 orders of magnitude (down to 10 fM within 30 min), compared to common hybridization assays [198]. MoS_2_ nanomaterials have also been used as photoactive nanomaterial, achieving very good visible light absorption performance and high transfer efficiency. More specifically, MoS_2_-AuNPs were synthesized on an electrode surface for the photoelectrochemical detection of miRNA. A biotinylated DNA probe was covalently immobilized on the nanosheets. Subsequently, miRNA was hybridized to the complementary DNA probe resulting in a signal increase. The protein streptavidin that binds to biotin moieties was also used for signal enhancement, achieving an LOD of 4.2 fM [199]. Similarly, a new electrochemical sensing platform was constructed to detect miRNA based on MoS_2_ nanosheets functionalized with thionine and AuNPs. Thionine was used as the reducing agent for current measurements. A DNA probe was immobilized onto this surface. After hybridization of the target miRNA to the probe, the duplex hybrid hindered the reduction of thionine, causing an electrochemical signal decrease. The LOD of the method was 0.26 pM [200].

Poly-xanthurenic acid-functionalized MoS_2_ nanosheets were also used for the electrochemical detection of ctDNA. A DNA probe was physically absorbed onto the nanosheets attached to an electrode. Target DNA was then hybridized to the probe and the hybrid was released from the surface of the nanosheets, leading to a signal increase that was measured by cyclic voltammetry and electrochemical impedance spectroscopy. The detection limit of this method was 18 aM [193]. An electrochemical biosensor was also constructed for ctDNA detection based on thin-layer MoS_2_/graphene nanosheets. A DNA specific probe was immobilized onto the sensor’s surface, while K_3_[Fe(CN)_6_] was used as the electroactive agent to monitor the hybridization of target miRNA by a signal decrease. The sensor had an LOD of 100 aM [201].

### Metal–organic frameworks (MOFs)

Metal–organic frameworks are a large family of materials with organic ligand-linkers and metal ions as nodes. They are crystalline materials which may form 3D structures with unique properties mainly electrical conductivity and quenching capability, high-pore volume and surface area and high thermal stability, possessing also graphene-like properties. MOFs can be attached to several biomolecules or other nanomaterials via electrostatic forces or covalent bonding. Their combination with other functional nanomaterials provided better performance than bare MOFs due to synergetic effects [4]. Copper-based metal–organic frameworks modified with AuNPs and DNA probes were constructed for miRNA detection based on SDA upon hybridization of the target miRNA to a complementary DNA hairpin probe immobilized on an electrode’s surface. Target miRNA was then displaced by a second hairpin, while the complex of AuNPs-MOFs was hybridized to a complementary segment of the second hairpin. Glycose was finally oxidized by the AuNPs-MOFs and an electrical signal was generated. The method had an LOD of 0.25 fM [202]. A GO-like fluorescent sensing system was also developed for miRNA detection using fluorescent ssDNA probes that were quenched by Mn-based MOFs with an LOD of 0.2 pM (Fig. 5c) [203], while a similar approach was reported using labeled specific PNA probes with different fluorophores for multiplex detection of miRNA molecules, providing an LOD of 10 pM (Fig. 5c) [204].

### Polymer nanoparticles (PNPs)

Although polymeric nanostructures have been widely utilized for drug delivery purposes, there are few reports for their exploitation in liquid biopsy tests. Carbonyl functioned poly(9,9-di-n-octylfluorenyl-2,7-diyl) (PFO) polymer nanoparticles were used for the ratiometric electrochemiluminescent determination of miRNA with a very low LOD of 17 aM in PBS buffer. Polyfluorene materials provide an excellent support that increase the quantum yield and the photostability of the compounds, along with easy bioconjugation and excellent ECL performance. In this approach, the PNPs were coupled to the hairpin H1 and were assembled onto a glassy carbon electrode. MiRNA target initiated an SDA reaction that produced a secondary target, which finally opened the hairpin H1. The two other hairpins tagged to glycose oxidase triggered an HCR onto the electrode’s surface, that upon H_2_O_2_ addition, led to increased ECL signal derived from the PNPs [205]. Another application involved the construction of a micro/nanostructure polymeric surface for CTCs selective enrichment. The authors succeeded cancer cell enrichment ratios about 5–8 times higher than those provided by untreated surface after a 3-day culture procedure [206]. Absorbent polymer beads (poly(acrylamide-co-acrylic acid)) were also used to provide concentration of exosomes with high purity [207]. Finally, cationic lipid-polymer hybrid nanoparticles were utilized in combination with CHA for the detection of extracellular vesicles (EVs) (LOD = 37.5 particles/mL) [208].

### Other nanoparticles

Several other nanoparticles have been utilized in assays for biomarker detection. Luminescent upconverting nanoparticles were used in combination with luminescence energy transfer by a molecular beacon, which was hindered after miRNA hybridization. This system offered an LOD 762 aM and an analysis time of 10 min [209]. The excellent catalytic properties of ZnO nanostars were also exploited along with a luminol-O_2_ system for an ultrasensitive electrochemiluminescent detection of microRNA (LOD 18.6 aM) [210]. Moreover, black phosphorus nanosheets [211] and MnO_2_ nanosheets [212] were used as new fluorescence quenching materials for the detection or imaging of miRNA with an LOD of 9.37 nM for the first approach and 9.8 pM for the second. Silica nanofibers incorporated to calcium fluoride particles (CaF_2_) and AuNPs were constructed as a novel FRET biosensor for miRNA detection (LOD was 2 nM) [213]. MiRNA molecules were also detected using a polydopamine nanosphere-assisted chemiluminescence resonance energy transfer (CRET) with an aid of a DSN-assisted signal amplification assay, offering an LOD of 49.6 pM [214]. An electrochemical biosensor based on polyethylene glycol (PEG)-polypyrrole nanowires and tungsten diselenide (WSe_2_) nanosheets-modified electrode were developed for the detection of as low as 33 fM and 0.06 fM of miRNA, respectively [215, 216]. Finally, novel multifunctional fluorescent SnO_2_ nanoparticles were constructed for the recognition of intracellular miRNAs [217].

The excellent conductivity and catalytic properties of two-dimensional Ti_3_C_2_ MXenes nanosheets were exploited for the construction of an aptamer- and luminol-based electrochemiluminescent biosensor for the detection of as low as 125 exosomes/μL, which was over 100 times lower than that of conventional ELISA method [218]. The luminescence resonance energy transfer from upconversion nanoparticles to gold nanorods was also reclaimed for the aptamer-based detection of exosomes with an LOD of 1.1 × 10^3^ particles/μL [219]. Exosomes and encapsulated RNA molecules were also detected by tethered cationic lipoplex nanoparticles containing molecular beacons [220]. Moreover, CTCs were detected (LOD 0.1 μM) by micellar nanoparticles that responded rapidly to the high level of endogenous H_2_O_2_ of CTCs through fluorescence emission [221] or by combination of X-rays with magnetic and bismuth nanoparticles with a detection limit of approximately 100 CTCs/mL [222].

## Discussion

Compared to the conventional methods, the use of nanomaterials has offered many advantages for the detection of specific biomarkers, such as miRNAs, circulating tumor (ctDNA) or cell-free DNA, circulating tumor cells (CTCs) and exosomes in liquid biopsy applications. The use of nanomaterials has greatly increased the detectability of novel approaches, as the low abundance of these biomarkers in blood circulation constitutes a significant challenge for analysts. A comparison of all nanomaterials and detection strategies developed, in terms of detectability (LOD), dynamic range, analysis time, amplification step (apart from the nanomaterials used) and multiplex application, are presented in Tables S1-S4 in the Additional file 1. All the methods presented in the comparative Tables have been applied to real sample analysis.

Gold nanoparticles have attracted scientific interest and have been exploited in most applications, as they allow for fast, simple, and sensitive detection, as well as exhibiting multi-functional properties. Moreover, the unique properties of iron magnetic nanomaterials, for example, their electrochemical, plasmonic and enzyme-like activity, have led to their use in a very wide range of applications. Moreover, the synthesis of such porous nanomaterials increases the surface-to-volume ratio that is crucial for signal enhancement. However, the best performance in terms of detectability was achieved, on one hand, by combining two or three different nanomaterials, thereby exploiting the alternative properties of the nanomaterials and leading to synergetic effect on signal generation, and, on the other hand, by introducing other signal amplification or target recycling steps based on hybridization chain reaction (HCR), catalytic hairpin assembly (CHA), rolling circle amplification (RCA), strand displacement amplification (SDA), duplex-specific nuclease (DSN), and DNAzyme. Most of these approaches required a prolonged analysis time-period for ultra-sensitive detection. Compared to other amplification strategies, HCR has significant advantages, which include low background, cost-effectiveness and better stability.

Nanomaterials have been used in electrochemical sensors as labels and signal enhancement agents. Electrochemical methods are the ones mostly used in this direction, mainly due to their low cost, fast analysis, easy operation, portability, high simplicity, sensitivity, and selectivity, as well as multiplicity potential. Several nanomaterials have emerged for signal enhancement. They have dramatically improved detectability by increasing the surface reacting area and accelerating the electron transfer between the reaction parties. Photoelectrochemical methods have provided us with excellent detectability and sensitivity, because of their low background signal. Further advantages arise from their low operation cost, easy operation, rapid analysis and fast response.

Furthermore, lateral flow assays (strips) are portable and simple to apply in a user-friendly format, while also providing rapid analysis with great detectability results. They can have also a significant contribution to multi-analyte potential based on spatial or spectral discrimination.

SERS- and SPR-based methods provide label-free detection. However, SERS has limitations due to the low reproducibility of the available SERS substrates. Nanoparticles or novel nanostructures have been used here as major signal enhancers as they improve reproducibility dispensing with the need for enzymatic reactions, sequence-specific enhancers or multiple enhancement steps. Metal nanoparticles have shown increased SERS and SPR or LSPR activity. The assessment of magnetic nanoparticles with SERS- or SPR-active metal nanoparticles resulted in enhanced signal production. Moreover, SERS has a narrow spectral bandwidth that can be exploited in multiplex analysis. Both techniques have excellent detectability and specificity, while multiplex analysis has been also reported. Blood samples, however, have strong interferences that limit their applications to liquid applications and further work is required in this direction. In addition, detecting single-point mutations is still challenging for these methods. Finally, single-molecule detection was achieved with the use of ultra-sensitive SERS-based methods.

Most of the techniques avoid the PCR amplification step in nucleic acid analysis, to the sacrifice—with few exceptions—of the selectivity of the method in case of 1–3 nucleotide mismatches in nucleic acids sequences. However, the analysis of irrelevant targets in all reported methods, has attained high selectivity and specificity. Specific CTCs and exosomes were also identified with high selectivity by means of the proposed methods.

Isothermal amplification techniques, without PCR, is the key for nucleic acids analysis in the majority of the reported methods. As for exosomes’ detection, several techniques are available. Electron microscopy provides information about the size and the shape of exosomes but does not allow for quantitative analysis. ELISA is used for the detection of specific exosomal proteins, albeit requiring expensive labelling of corresponding antibodies. Nanoparticle tracking analysis (NTA) is the most recent technique based on light scattering of the analyzed exosomes, but has a narrow working range (10^6^–10^9^ exosomes/mL) and is limited to multiplex analysis. Flow cytometry has a good analytical performance, but requires expensive instrumentation and large sample volumes. Exosomes contain several kinds of proteins that can be targeted by specific antibodies or aptamers. The use of specific aptamers, however, is the most attractive alternative as it reduces the cost of the analysis. Aptamers are preferred in most applications, given that they have very good specificity and binding affinity to exosomes. In addition, the combination of aptamers with various nanoparticles has also enhanced the signal to the effect of detecting exosomes in extremely low concentrations.

The most sensitive techniques reported for liquid biopsy applications were the fluorometric and electrochemical ones, as well as the SERS- and SPR-based methods, while the majority of the colorimetric methods, apart from the lateral flow assays, revealed quite low detection limits. Colorimetric methods are the most convenient, but provided poor detectability, which limits their applications in cancer diagnostics. Electrochemical-based methods have gained the interest of researches due to the cost-effective instrumentation, automation, high sensitivity and fast analysis, yet they lack high specificity in point-mutation detection. A comparison of all the nanomaterials used for liquid biopsy testing with regard to the lowest LOD achieved, is presented in Table 1. Silver nanoparticles, especially in combination with AuNPs or other metal nanomaterials, achieved the best performance compared to the other nanomaterials, for all the biomarkers under discussion. SPR and SERS, along with fluorescence-based methods, have proven to be very useful analytical techniques for ultrasensitive detection of biomarkers. Nanomaterials have extensively served as signal enhancers in SPR and SERS. Single-molecule detection of miRNA was achieved by developing an SPR sensor using gold nanoparticles for signal enhancement. Amplification-free electrochemical, SPR and SERS methods have also been developed with good detectability. Ultrasensitive methods, achieving extremely low LODs were also reported. More specifically, fluorescent carbon dots and MnO_2_ nanosheets were successfully used for the detection of miRNA, giving an LOD of 0.1 aM, while 10 aM of ctDNA were detected by a molybdenium disulfide-based nanomaterial. Moreover, a single CTC was detected using either QDs and fluorescence microscopy or silver and magnetic nanoparticles combined with SERS signaling. Finally, Raman spectroscopy with gold nanoparticles and fluorescence-based methods with GO or cationic lipid-polymer nanoparticles proved to be ideal for exosome analysis. All the above-mentioned nanomaterials exhibited good analytical performance providing a wide dynamic range and extremely low LODs at the attomolar level, with the exception of silica nanoparticles, in which case only one report offered LOD in the femtomolar level. Silica nanoparticles, however, are preferred in many reports due to their low cost and ease of synthesis. All the nanomaterials discussed here have proven suitable for real samples analysis without necessitating a complicated pretreatment of the samples. However, many of the methods reported have to still be applied to real-sample analysis to evaluate their specificity and detectability. The drawbacks for many systems lie to extensive fabrication and analysis steps, that hinder routine analysis testing. Lateral flow assays are superior towards this direction. In conclusion, other parameters such as the cost and the simple synthesis of the nanomaterial, the sample size, the specificity, the biocompatibility, the portability, the simple conjugation procedure to various biomolecules, the application to real samples, as well as the time and the total cost of the analysis have to be carefully considered in order to establish the optimum nanomaterial for specific biomolecular sensing. Further effort is still required for the development of portable point-of-care devices with a simple, easy and user-friendly format, while nanomaterials-based miniaturized devices will definitely be the pioneers in this venture.

**Table 1:** Overview of the methods with the best analytical performance for the detection of miRNA, ctDNA, CTCs and exosomes using various nanoparticles/nanomaterials

Nanoparticles	Lowest limit of detection (LOD)/Method used			
	*miRNA*	*ctDNA*	*CTCs*	*Exosomes*
Gold nanoparticles	0.33 aM, 1 aM—0.1 nM graphdiyne decorated with AuNPs photoelectrochemistry 6.8 aM, 10 aM—10 pM DSN amplification electrochemistry single-molecule detection, SPR	100 aM, 0.1–10 fM PCR amplification lateral flow assay	10 CTCs (10 cells/mL) 10–100 cells/mL RCA amplification ICP-MS	32 exosomes/μL 1.25 × 10^5^–1.25 × 10^9^ exosomes/mL SERS
Graphene oxide	10 aM, 50 aM–5 fM DSN amplification pulse voltametry	–	2CTCs/4 mL, immunostaining	50 exosomes/μL 10^6^–10^9^ exosomes/mL fluorescence
Other carbon nanomaterials	0.1 aM, 0.15–20 aM + AuNPs, fluorescence 0.3 aM, 1 aM–0.1 μM + MnO_2_ nanosheets fluorescence	0.3 fM, 10 fM–1 nM RNase HII amplification SWCNTs, SERS	2 CTCs (20 cells/mL) 10^2^–10^6^ cells/mL electrochemiluminescence	5.2 × 10^5^ exosomes/μL 1.84 × 10^9^–2.21 × 10^10^ exosomes/mL colorimetric
Quantum dots	17 aM, 50 aM–50 pM DSN amplification photoelectrochemistry	–	1 CTC, fluorescence microscopy 1 CTC, fluorescence (QDs-magnetic NPs)	100 exosomes/μL 10^5^—10^10^ exosomes/mL stripping voltametry
Copper nanoparticles	10 aM, 0.1 fM–10 pM HCR, SDA and T7 exonuclease amplification electrochemistry	–	–	4.8 × 10^4^ exosomes/μL 7.5 × 10^7^–1.5 × 10^10^ exosomes/mL fluorescence
Silver nanoparticles	1 aM, 1 aM–1 nM Au/Ag nanocube, LSPR	40.4 aM, 1 fM–1 μM HCR amplification SERS	1 CTC/2 μL (6 cells/mL) 50–10^5^ cells/mL Chriplasmonic 1 CTC/mL 10–10^3^ cells/mL Ag-iron oxide NPs, SERS	1 exosome/2 μL 5.4 × 10^2^–2.7 × 10^10^ exosomes/mL SERS
Magnetic (iron) nanoparticles	10 aM, 10 aM–10 nM gold-coated magnetic nanoparticles electrochemistry	-	1 CTC/mL 1–500 cells/mL SERS	100 exosomes, magnetic resonance imaging
Silica nanoparticles	30 fM, 0.9–10 pM electrochemistry	-	–	–
Molybdenium disulfide nanomaterials	30 aM, 0.1 fM–10 nM field-effect transistor	18 aM, 0.1 fM–0.1 nM electrochemistry	–	–
Metal–organic frameworks	350 aM, 1 fM–10 nM AuNPs-MOFs SDA amplification electrochemistry	–	–	–
Polymer nanoparticles	17 aM, 50 aM–100 pM polyfluorene polymer nanoparticles electrochemiluminescence	–	–	37.5 exosomes/mL 0.18–3.0 × 10^6^ exosomes/mL cationic lipid-polymer nanoparticles fluorescence
Other nanoparticles				
*Tungsten diselenide nanosheets*	60 aM, 0.1 fM–100 pM DSN amplification electrochemistry	–	–	–
*ZnO nanostars*	18.6 aM, 100 aM–100 pM electrochemiluminescence	–	–	–
*Bismuth nanoparticles*	–	–	100 CTCs/mL 10^2^–10^5^ cells/mL X-rays	–

## Conclusions

A wide range of nanoparticles and nanomaterials that are used in liquid biopsy applications has been reported in this review. Compared to conventional methods, these nanomaterials provide high sensitivity and rapid analysis. The properties of nanoparticles are size-dependent and have to be carefully optimized for excellent performance. Regarding liquid biopsy applications, great progress has been achieved for enhanced sensing systems development, while significant efforts improved the analytical performance. Signal enhancement protocols were based on target recycling and enzyme amplification in combination with novel nano-architectures that increase the signal and succeed target enrichment. The use of nanomaterials enhanced the capture efficiency, mainly due to the large surface-to-volume ratio, increasing the detectability. The combination of specific bio-recognition molecules, such as aptamers, peptides and DNA/PNA probes, with novel nanomaterials greatly improved the detection efficiency. Finally, lots of successful clinical application have been achieved, ensuring liquid biopsy as promising non-invasive analytical tool in routine clinical diagnostics.

Liquid biopsy is still at the early stages of its development. Extensive work is required to increase the robustness and method standardization, in order to integrate nanomaterials to portable point-of-care sensing devices. Currently, liquid biopsies only serve as supportive information to the traditional methods. Only two methods or diagnostic kits have hitherto been granted FDA approval: (i) a real-time-based kit for the detection of seven specific mutations in epidermal growth factor receptor (EGFR) gene in ctDNA and (ii) a fluorescent immunomagnetic test kit for CTCs isolation and identification [223, 224].

Future perspectives for precision oncology are currently turned to “multiomics” analysis, i.e., the simultaneous analysis of multiple different kinds of biomarkers. This will lead to more detailed information about tumor heterogeneity and metastasis. Nanomaterials have a huge potential in this direction and, consequently, in liquid biopsy applications in particular.

## Supplementary Information

**Additional file 1.** Additional tables.

## Data Availability

Not applicable.
