# Supplementary material for: Nanotechnology in emerging liquid biopsy applications
Source: Nano Converg. 2021 May 2;8:13. doi: 10.1186/s40580-021-00263-w (PMC8088419; doi:10.1186/s40580-021-00263-w)
Supplement: Supplementary file 1 — Additional file 1. Additional tables. [file 40580_2021_263_MOESM1_ESM.docx]

**Nanotechnology in emerging liquid biopsy applications**

Despina P. Kalogianni

Corresponding Author

Despina P. Kalogianni

kalogian@upatras.gr

Department of Chemistry, University of Patras, Rio, Patras 26504, Greece

**Table S1: miRNAs**

| **Nanomaterial** | **Technique** | **Amplification strategy** | **Dynamic Range** | **LOD** | **Analysis time** | **Multiplicity** | **Ref.** |
| --- | --- | --- | --- | --- | --- | --- | --- |
| ***Gold nanoparticles*** | Colorimetric | CHA and nicking endonuclease | 1.3 fM – 1.3 nM | 3.1 fM | - | - | 39 |
|  | Colorimetric | - | 1 – 250 pM | 1.67 pM | > 3 h | - | 71 |
|  | Colorimetric (universal) |  | 0.17 – 0.83 μM | 0.33 μM | few minutes | - | 40 |
|  | Colorimetric | - | 100 aM – 100 fM | **100 aM** | 1 h | - | 41 |
|  | LFA* | - | 0.01 – 5 nM  0.05 – 10 nM | 7 pM | 10 min | 3 | 29 |
|  | LFA | RCA* | 2 pM - 200 nM | 20 pM | 15 min | 2 | 42 |
|  | LFA | - | 0 – 75 nM | 7.5 pM | 30 min | - | 43 |
|  | Fluorescence (universal) | DNAzyme and click chemistry | 1 pM – 10 nM | 50 fM | 1 h | - | 32 |
|  | Fluorescence (universal) | - | 10 – 100 pM | 8.4 pM | > 17 h | - | 70 |
|  | Colorimetric and fluorescence | - | 20 – 100 pM | 4.4 pM | 2 h | - | 38 |
|  | Electrochemistry | HCR* | 1 fM – 1 nM | **120 aM** | 2.5 h | - | 26 |
|  | Electrochemistry | - | 500 aM – 1 μM | **135 aM** | > 2 h | - | 28 |
|  | Electrochemistry | HCR and RNA endonuclease | 0.1 pM – 10 nM | 100 fM | 5.5 h | - | 48 |
|  | Electrochemistry | - | 100 aM – 1 nM | **78 aM** | 3.5 h | - | 49 |
|  | Electrochemistry | DSN* | 10 aM – 10 pM | **6.8 aM** | > 3 h | - | 51 |
|  | Electrochemistry | Silver enhancement | 10 fM – 1 nM | 4 fM | 3 h | - | 52 |
|  | Electrochemistry | DSN | 0.1 fM – 100 pM | **43.3 aM** | 3 h | - | 53 |
|  | Electrochemistry | DSN | 1 pM – 100 nM | 170 fM | > 2.5 h | - | 54 |
|  | Electrochemiluminescence | - | - 1. fM – 0.1 μM | **43 aM** | > 4h | - | 46 |
|  | Electrochemiluminescence | - | 10 fM – 1 nM | 10 fM | > 30 min | - | 50 |
|  | Photoelectrochemistry | - | 5 – 50 nM | 2 nM | 1 h | - | 47 |
|  | Photoelectrochemistry | - | 0.5 fM – 5 pM | **130 aM** | 1 h | - | 44 |
|  | Photoelectrochemistry | - | 1 aM – 0.1 nM | **0.33 aM** | 3.5 h | - | 45 |
|  | SPR* | - | 0.5 pM – 1 nM | 0.5 pM | > 30 min | 4 | 31 |
|  | SPR | - | 100 nM – 1 μM | 100 nM | - | - | 56 |
|  | SPR | - | 447 pM – 4 nM | 447 pM | > 1 h | - | 57 |
|  | SPR | - | 1 pM – 100 nM | 270 fM | 2.5 h | - | 58 |
|  | SPR | SDA* | 50 pM – 5 mM | 45 pM | 2.5 h | - | 60 |
|  | LSPR* | - | 10 pM – 1 μM | 5 pM | > 2 h | - | 61 |
|  | SPR | - | 0 – 50 pM | **0.5 fM** | 2 h | - | 62 |
|  | SPR | HCR and CHA* | 50 aM – 2 fM | **50 aM** | 5 h | - | 63 |
|  | LSPR | - | 0.1 fM – 10 nM | **83 aM** | overnight | - | 64 |
|  | SERS* | - | 0.1 fM – 1 μM | 2.16 fM | 18 h | 3 | 65 |
|  | SERS | - | 50 aM – 500 fM | **50 aM** | - | - | 66 |
|  | SERS and electrochemistry | - | 10 pM – 450 nM | 100 fM | - | - | 67 |
|  | SERS | - | 1 fM – 10 pM | **700 aM** | 4 h | - | 68 |
|  | QCM* | - | 10 pM – 1 nM | 10 pM | > 3 h | - | 69 |
|  | Dark-field microscopy | SDA | 4 pM – 20 nM | 13.5 fM | 40 min | - | 55 |
| ***Graphene oxide*** | Fluorescence (universal) | RCA | 1 fM – 10 mM | **870 aM** | 4 h | 2 | 92 |
|  | Fluorescence (universal) | RCA | 0 – 100 nM | 250 pM | > 4 h | 2 | 93 |
|  | Fluorescence | HCR | 10 fM – 2 pM | 4.2 fM | 50 min | - | 94 |
|  | Fluorescence (universal) | CHA | 0 – 16 nM | 47 pM | > 3 h | - | 95 |
|  | Fluorescence | - | 1 – 50 nM | 180 pM | > 30 min | - | 99 |
|  | Fluorescence (universal) | - | 0 – 1 μM | 1 pM | 14 h | 3 | 101 |
|  | Fluorescence | HCR | 0.1 – 10 nM | 1.74 nM | 6 h | - | 111 |
|  | Fluorescence | - | 1 pM – 1 nM | 1 pM | - | 2 | 102 |
|  | Electrochemistry | - | 10 pM – 10 mM | 1 pM | > 3 h | - | 33 |
|  | Electrochemistry | HCR and CHA | 10 fM – 5 nM | 1.57 fM | 4.5 h | - | 37 |
|  | Electrochemistry | DSN | 50 aM – 5 fM | **10 aM** | > 5 h | - | 105 |
|  | Electrochemistry | - | 10 fM – 10 nM | 1.5 fM | > 1 h | - | 90 |
|  | Electrochemistry | DSN | 10 aM – 0.1 nM | **34 aM** | > 2 h | - | 107 |
|  | Electrochemistry | - | 1 fM – 1 μM | 1 fM | 30 min | - | 108 |
|  | Electrochemistry | DSN | 1 fM – 10 pM | 1.5 fM | 80 min | - | 109 |
|  | Electrochemistry | - | 10 fM – 1 nM | **170 aM** | > 3 h | - | 110 |
|  | Electrochemistry | CHA | 10 fM – 100 pM | 4.3 fM | 1 h | - | 112 |
|  | Electrochemistry | - | 1.4 – 7 μM | 700 pM | 1 h | - | 115 |
|  | Electrochemiluminescence | HCR | 0.1 fM – 1 pM | **30 aM** | 2 h | - | 97 |
|  | SPR | - | 10 fM – 10 pM | **100 aM** | 1 h | - | 106 |
| ***Carbon nanomaterials*** |  |  |  |  |  |  |  |
| CQDs | Fluorescence | - | 5 – 160 nM | 300 fM | 20 min | - | 7 |
| MWCNTs | Fluorescence (universal) | DSN | 100 fM – 1 nM | 33.4 fM | 2.5 h | - | 8 |
| SWCNTs | Fluorescence (universal) | - | 10 pM – 1 μM | 10 pM | 1 h | 2 | 87 |
| Carbon nitride nanosheets | Fluorescence (universal) | HCR | 1 – 800 pM | 200 fM | 4 h | - | 89 |
| Carbon nanotubes | Fluorescence | - | 0.01 – 100 nM | 36 pM | 40 min | - | 123 |
| Carbon dots/AuNPs | Fluorescence | - | 1 aM – 0.1 μM | **0.3 aM** | 20 min | - | 133 |
| Carbon dots/MnO_2_ nanosheets | Fluorescence | - | 0.15 – 20 aM | **0.1 aM** | > 10 min | - | 134 |
| SWCNTs | Electrochemistry (universal) | Exonuclease | 0.01 – 100 pM | 3.5 fM | 2 h | - | 11 |
| SWCNTs | Electrochemical impedance spectroscopy | - | 1 pM – 10 nM | 300 fM | - | - | 124 |
| Carbon nanospheres | Electrochemistry | CHA | 0.1 fM – 0.1 nM | **16 aM** | 3 h | - | 125 |
| Carbon nanospheres | Electrochemistry | - | 0.1 fM – 0.1 nM | **100 aM** | - | 2 | 126 |
| Carbon black  MWCNTs and GO | Electrochemistry | - | 1 nM – 2 μM | 10 pM | > 1h | - | 127 |
| Carbon nanofibers | Electrochemistry | - | 35 – 140 μM | 1.5 μM | 20 min | - | 128 |
| SWCNTs and nanodiamonds | Electrochemistry | HCR and DNAzyme | 10 fM – 1 nM | 1.95 fM | 3 h | - | 129 |
| Nanodiamonds | Electrochemistry | RCA, DSN and exonuclease III | 0.25 fM – 0.25 nM | **83.3 aM** | 6.5 h | - | 131 |
| CQDs | Photoelectrochemistry | DSN | 0.5 fM – 5 pM | **150 aM** | 1 h | 2 | 130 |
| ***Quantum dots*** | LFA | SDA | 0.1 – 10 nM | 10 pM | 80 min | - | 142 |
|  | Fluorescence | - | 5 – 150 nM | 380 pM | 45 min | - | 141 |
|  | Fluorescence | - | 5 pM – 50 nM | 1.2 pM | 1 h | - | 148 |
|  | Fluorescence | Exonuclease III | 5 pM – 50 nM | 1.5 pM | 6.5 h | 2 | 149 |
|  | Electrochemistry | DSN | 0.1 – 100 pM | 33 fM | 4 h | - | 143 |
|  | Electrochemiluminescence (universal) | SDA | 1 fM – 100 pM | **280 aM** | 20 min | - | 136 |
|  | Electrochemiluminescence | - | 10 fM – 100 pM | 10 fM | 1 h | - | 146 |
|  | Electrochemiluminescence | - | 1 fM – 10 nM | **200 aM** | 1 h | - | 147 |
|  | Photoelectrochemistry | SDA | 0.1 fM – 10 nM | **49 aM** | - | - | 137 |
|  | Photoelectrochemistry | - | 1 fM – 10 pM | **500 aM** | overnight | - | 138 |
|  | Photoelectrochemistry | - | 20 fM – 0.2 nM | 5.6 fM | > 1 h | - | 139 |
|  | Photoelectrochemistry | DSN | 50 aM – 50 pM | **17 aM** | 40 min | - | 140 |
|  | Photoelectrochemistry | - | 1 fM – 10 pM | 1 fM | > 2.5 h | 2 | 144 |
|  | Flow cytometry | Exonuclease VII | - | - | - | **12** | 150 |
| ***Copper nanomaterials*** | Colorimetric | - | 1 pM – 10 nM | 600 fM | 2 h | - | 156 |
|  | Fluorescence | DSN | 20 – 1000 pM | 20 pM | > 6.5 h | - | 157 |
|  | Fluorescence and electrochemistry | - | 0 - 100 pM | 100 fM | 10 min fluorescence  45 min EC | - | 158 |
|  | Fluorescence | - | 5 pM – 10 nM | 2.2 pM | 6.5 h | - | 161 |
|  | Fluorescence | Klenow Fragment exonuclease | 1 pM – 1 nM | 100 fM | 3 h | - | 163 |
|  | Electrochemistry | HCR, SDA and T7 exonuclease | 0.1 fM – 10 pM | **10 aM** | > 6.5 h | - | 162 |
|  | Electrochemiluminescence | SDA, T7 exonuclease and phi29 polymerase | 100 aM – 100 pM | **36 aM** | 5.5 h | - | 159 |
|  | Electrochemiluminescence | HCR and exonuclease III | 100 aM – 100 pM | **19.05 aM** | 3.5 h | - | 160 |
| ***Silver nanoparticles*** | Colorimetric | - | 1 nM – 5 μM | 600 pM | 1.5 h | - | 165 |
|  | Fluorescence | - | 1 pM – 5 μM | 400 fM | 1.5 h | - | 165 |
|  | Fluorescence | CHA | 0 – 200 nM | 300 fM | 75 min | - | 164 |
|  | Fluorescence | DSN | 100 fM – 1 nM | 33.4 fM | 85 min | - | 166 |
|  | Electrochemistry | - | 0.1 fM – 50 fM | **20 aM** | 1.5 h | - | 168 |
|  | SERS | - | 1 pM – 100 nM | 1 pM | 15 min | - | 169 |
|  | LSPR | - | 1 aM – 1 nM | **1 aM** | 3 h | - | 167 |
| ***Silica nanoparticles*** | Fluorescence | - | 0.5 – 20 nM | 160 pM | 8 h | - | 175 |
|  | Fluorescence | HCR, CHA and DNAzyme | 10 pM – 2 nM | 2.5 pM | 3.5 h | - | 176 |
|  | Photoluminescence | - | 50 – 500 nM | 2 nM | - | - | 177 |
|  | Electrochemistry | - | 0.9 – 10 pM | 30 fM | > 3 h | - | 37 |
| ***Iron/magnetic nanoparticles*** | Electrochemistry | - | 100 aM – 1 nM | **100 aM** | > 1.5 h | - | 181 |
|  | Electrochemistry | - | 10 aM – 10 nM | **10 aM** | 1 h | - | 182 |
|  | Electrochemistry | - | 1 fM – 1 nM | 1 fM | - | - | 183 |
|  | SERS | - | 100 fM – 1 nM | 100 fM | 2 h | - | 179 |
|  | SERS | - | 1.8 pM – 1.8 nM | 1.8 pM | > 2 h | - | 180 |
| ***MoS_2_ nanomaterials*** | Fluorescence | - | 0 – 40 nM | 500 pM | 40 min | - | 195 |
|  | Fluorescence | - | 10 – 50 nM | 10 nM | 2 h | - | 196 |
|  | Fluorescence | DSN | 10 fM – 10 nM | 10 fM | 50 min | - | 200 |
|  | Electrochemistry | - | 1 fM – 0.1 nM | **430 aM** | > 40 min | - | 194 |
|  | Electrochemistry | - | 1 pM – 10 nM | 260 fM | 50 min | - | 192 |
|  | Photoelectrochemistry | - | 10 fM – 1 nM | 42 fM | 2.5 h | - | 201 |
|  | Field-effect transistor | - | 0.1 fM – 10 nM | **30 aM** | 18 h | - | 199 |
| ***MOFs*** | Fluorescence | HCR | 0.5 – 100 pM | 500 fM | 2-6 h | - | 203 |
|  | Fluorescence | - | 0 – 1000 nM | 10 pM | 2 h | 3 | 204 |
|  | Electrochemistry | SDA | 1 fM – 10 nM | **350 aM** | 2 h | - | 202 |
| ***Polymer nanoparticles*** | Electrochemiluminescence | SDA, HCR | 50 aM – 100 pM | **17 aM** | 7 h | - | 205 |
| ***Other nanoparticles*** |  |  |  |  |  |  |  |
| Luminescence upconversion nanoparticles | Luminescence | - | 1 fM – 1 pM | **762 aM** | 10 min | - | 209 |
| Polydopamine nanospheres | Chemiluminescence | DSN | 80 pM – 50 nM | 49.6 pM | 2h | - | 214 |
| Black phosphorus nanosheets | Fluorescence | - | 10 – 1000 nM | 9.4 nM | 30 min | - | 211 |
| MnO_2_ nanosheets | Fluorescence | HCR | 0 – 100 nM | 9.8 pM | 3.5 h | - | 212 |
| Calcium floride nanoparticles | Fluorescence | - | 2 – 500 nM | 2 nM | 1 h | - | 213 |
| Polypyrrole nanowires | Electrochemistry | - | 0.1 pM – 1 nM | 33 fM | 1 h | - | 215 |
| Tungsten diselenide nanosheets | Electrochemistry | DSN | 0.1 fM – 100 pM | **60 aM** | 2 h | - | 216 |
| ZnO nanostars | Electrochemiluminescence | - | 100 aM – 100 pM | **18.6 aM** | 4.5 h | - | 210 |

^*^ LFA: Lateral flow assay

SERS: Surface-enhanced Raman spectroscopy

SPR: Surface plasmon resonance

LSPR: Localized Surface plasmon resonance

QCM: Quartz crystal microbalance

HCR: Hybridization chain reaction

CHA: Catalytic hairpin assembly

RCA: Rolling circle amplification

SDA: Strand displacement amplification

DSN: Duplex-specific nuclease

**Table S2: Circulating tumor DNA (ctDNA)**

| **Nanomaterial** | **Technique** | **Amplification strategy** | **Dynamic Range** | **LOD** | **Analysis time** | **Multiplicity** | **Ref.** |
| --- | --- | --- | --- | --- | --- | --- | --- |
| ***Gold nanoparticles*** | Colorimetric | CHA | 870 aM – 87 pM | 7.7 fM | - | - | 75 |
|  | LFA (universal) | - | 0.1 – 10 fM | **100 aM** | 5 min | - | 77 |
|  | SERS | - | 100 – 1000 nM | 57.74 nM | 1 h | - | 74 |
|  | LSPR | - | 50 fM – 3.2 pM | 200 fM | 30 min | 2 | 73 |
| ***Carbon nanomaterials*** |  |  |  |  |  |  |  |
| SWCNTs | SERS (universal) | RNase HII | 10 fM – 1 nM | **300 aM** | - | - | 135 |
| ***Silver nanoparticles*** | SERS | HCR | 1 fM – 1 μM | **40.4 aM** | 3 – 6 h | - | 170 |
| ***Iron/magnetic nanoparticles*** | Electrochemistry | - | 0 – 1000 copies | 50 copies | 30 min | - | 185 |
|  | ICP-MS^*^ |  | 0.06 – 0.3 ng/mL | 0.1 pg/mL | - | - | 184 |
| ***MoS_2_ nanomaterials*** | Electrochemistry | - | 0.1 fM – 0.1 nM | **18 aM** | - | - | 193 |
|  | Electrochemistry | - | 0.1 fM – 0.1 pM | **100 aM** | 35 min | - | 201 |

^*^ICP-MS: Inductively coupled plasma mass spectrometry

**Table S3: Circulating tumor cells (CTCs)**

| **Nanomaterial** | **Technique** | **Amplification strategy** | **Dynamic Range** | **LOD** | **Analysis time** | **Multiplicity** | **Ref.** |
| --- | --- | --- | --- | --- | --- | --- | --- |
| ***Gold nanoparticles*** | LFA | - | 8×10^5^ - 4×10^7^ cells/mL | 1.6×10^5^ cells/mL | 25 min | - | 80 |
|  | ICP-MS | RCA | 10 – 100 cells/mL | **10 cells/mL** | > 6 h | - | 78 |
|  | LDI-MS^*^ | - | 50 - 5×10^4^ | 500 cells/mL | > 1.5 h | - | 79 |
| ***Graphene oxide*** | Fluorescence | Nicking endonuclease | 50 – 10^5^ cells/mL | 25 cells | > 2 h | - | 118 |
|  | Electrochemiluminescence | - | - | 40 cells/mL | 15 min | - | 119 |
|  | Field effect transistor (universal) | - | 10 – 10^6^ cells/mL | **10 cells/mL** | - | - | 117 |
| ***Carbon nanomaterials*** |  |  |  |  |  |  |  |
| Carbon nitride nanosheets/AuNPs | Electrochemiluminescence | - | 10^2^ – 10^6^ cells/mL | **20 cells/mL** | 3.5 h | - | 28 |
| ***Quantum dots*** | Fluorescence | - | 60 – 600 cells/mL | 60 cells/mL | 15 min | - | 151 |
|  | Fluorescence | HCR | - | Single cell | 20 min |  | 152 |
|  | Electrochemiluminescence | RCA | 10 – 10^4^ cells/mL | **10 cells/mL** | - | - | 153 |
| ***Silver nanoparticles*** | SERS | - | 10 – 10^3^ cells/mL | **1 cell/mL** | 30 min | - | 171 |
|  | Chiroplasmonic | - | 50 – 10^5^ cells/mL | **6 cells/mL** | - | - | 172 |
| ***Iron/magnetic nanoparticles*** | Fluorescence | - | 10^2^ – 10^5^ cells/mL | 100 cells/mL | 100 min | - | 187 |
|  | Fluorescence | - | 25 – 100 cells/mL | 25 cells/mL | 15 min | - | 188 |
|  | SERS | - | 1 – 500 cells/mL | **1 cell/mL** | > 35 min | - | 186 |
| ***Polymer nanoparticles*** | Cell staining | - | - | 5-8 times cell enrichment | 3-day culture | - | 206 |
| ***Other nanoparticles*** |  |  |  |  |  |  |  |
| Bismuth nanoparticles | X-rays | - | 10^2^ – 10^5^ cells/mL | 100 cells/mL | 10 min | - | 222 |

^*^LDI-MS: Laser Desorption/Ionization – Mass spectrometry

**Table S4: Exosomes**

| **Nanomaterial** | **Technique** | **Amplification strategy** | **Dynamic Range** | **LOD** | **Analysis time** | **Multiplicity** | **Ref.** |
| --- | --- | --- | --- | --- | --- | --- | --- |
| ***Gold nanoparticles*** | LFA (universal) | - | 14 -24 g/mL | 8.5×10^5^ exosomes/μL | 15 min | - | 86 |
|  | Fluorescence | HCR and DNA dendrimers | 1.75×10^3^ - 7×10^6^ exosomes/μL | 1.16×10^3^ exosomes/μL | > 3 h | - | 82 |
|  | SERS | - | 10^2^ - 10^4^ exosomes/μL | 100 exosomes/μL | 40 min | 4 | 27 |
|  | SERS | - | 10^3^ - 10^5^ exosomes/μL | 2×10^3^ exosomes/μL | 2 h | - | 83 |
|  | SERS | - | 1.25×10^2^ – 1.25×10^6^ exosomes/μL | **32 exosomes/μL** | 2 h | 3 | 84 |
|  | SPR | - | 0 – 10^5^ exosomes/μL | **5 exosomes/μL** | 3 h | - | 81 |
|  | LSPR | - | 0.194 – 100 μg/μL | 0.194 μg/μL | few sec | - | 85 |
| ***Graphene oxide*** | Colorimetric | - | 1.9×10^6^ – 4.35×10^7^ exosomes/μL | 1.3×10^6^ exosomes/μL | 30 min | - | 121 |
|  | Fluorescence | DNase I | 3×10^4^ – 6×10^5^ exosomes/μL | 2.1×10^4^ exosomes/μL | 40 min | - | 120 |
|  | Fluorescence | - | 10^3^ – 10^6^ exosomes/μL | **50 exosomes/μL** | - | - | 122 |
| ***Carbon nanomaterials*** |  |  |  |  |  |  |  |
| SWCNTs | Colorimetric (universal) | - | 1.84×10^6^ – 2.21×10^7^ exosomes/μL | 5.2×10^5^ exosomes/μL | 40 min | - | 88 |
| ***Quantum dots*** | Electrochemistry | - | 10^2^ - 10^7^ exosomes/μL | 100 exosomes/μL | > 2 h | - | 155 |
| ***Copper nanomaterials*** | Fluorescence | - | 7.5×10^4^ – 1.5×10^7^ exosomes/μL | 4.8×10^4^ exosomes/μL | 2 h | - | 30 |
| ***Silver nanoparticles*** | SERS |  | 1 exosome/ 2 μL – 2.7×10^7^ exosomes/μL | **1 exosome/ 2 μL** | 2.5 h | - | 164 |
| ***Iron/magnetic nanoparticles*** | Colorimetric | - | 10^3^ – 10^7^ exosomes/mL | 10^3^ exosomes/mL | 2 h | - | 191 |
|  | SERS | - | 2.3×10^2^ – 2.4×10^5^ exosomes/μL | 60 exosomes/μL | 1,5 h | - | 190 |
|  | MRI^*^ | - | - | **25 exosomes/μL** | 1,5 h | - | 189 |
| ***Polymer nanoparticles*** | Nanoparticle tracking analysis | - | - | cell enrichment | 30 min | - | 207 |
|  | Fluorescence | CHA | 0.18–3.0×10^3^ exosomes/μL | **37.5 exosomes/mL** | - | - | 208 |
| ***Other nanoparticles*** |  |  |  |  |  |  |  |
| Ti_3_C_2_ nanomaterial | Electrochemiluminescence | - | 5×10^2^ – 5×10^6^ exosomes/μL | 125 exosomes/μL | 2 h | - | 218 |
| Luminescence upconversion nanoparticles | Luminescence | - | 10^4^ – 10^8^ exosomes/μL | 1.1×10^3^ exosomes/μL | 1 h | - | 219 |

^*^MRI: magnetic resonance imaging
